# Conservation Priorities in a Biodiversity Hotspot: Analysis of Narrow Endemic Plant Species in New Caledonia

**DOI:** 10.1371/journal.pone.0073371

**Published:** 2013-09-18

**Authors:** Adrien S. Wulff, Peter M. Hollingsworth, Antje Ahrends, Tanguy Jaffré, Jean-Marie Veillon, Laurent L’Huillier, Bruno Fogliani

**Affiliations:** 1 Université de la Nouvelle-Calédonie (UNC), Laboratoire Insulaire du Vivant et de l’Environnement (LIVE-EA 4243), Nouméa, New Caledonia; 2 Institut Agronomique néo-Calédonien (IAC), Diversités biologique et fonctionnelle des écosystèmes, Païta, New Caledonia; 3 Royal Botanic Garden Edinburgh, Edinburgh, United Kingdom; 4 Institut de Recherche pour le Développement (IRD), UMR AMAP, Laboratoire de botanique et d’Ecologie végétale appliquées, Herbarium NOU, Nouméa, New Caledonia; Universidad Nacional Autonoma de Mexico, Mexico

## Abstract

New Caledonia is a global biodiversity hotspot facing extreme environmental degradation. Given the urgent need for conservation prioritisation, we have made a first-pass quantitative assessment of the distribution of Narrow Endemic Species (NES) in the flora to identify species and sites that are potentially important for conservation action. We assessed the distributional status of all angiosperm and gymnosperm species using data from taxonomic descriptions and herbarium samples. We characterised species as being NES if they occurred in 3 or fewer locations. In total, 635 of the 2930 assessed species were classed as NES, of which only 150 have been subjected to the IUCN conservation assessment. As the distributional patterns of un-assessed species from one or two locations correspond well with assessed species which have been classified as Critically Endangered or Endangered respectively, we suggest that our distributional data can be used to prioritise species for IUCN assessment. We also used the distributional data to produce a map of “Hotspots of Plant Narrow Endemism” (HPNE). Combined, we used these data to evaluate the coincidence of NES with mining activities (a major source of threat on New Caledonia) and also areas of conservation protection. This is to identify species and locations in most urgent need of further conservation assessment and subsequent action. Finally, we grouped the NES based on the environments they occurred in and modelled the habitat distribution of these groups with a Maximum Entropy Species Distribution Model (MaxEnt). The NES were separable into three different groups based primarily on geological differences. The distribution of the habitat types for each group coincide partially with the HPNE described above and also indicates some areas which have high habitat suitability but few recorded NES. Some of these areas may represent under-sampled hotspots of narrow endemism and are priorities for further field work.

## Introduction

New Caledonia is a global biodiversity hotspot [1], [2] and contains some 3371 native species of vascular plants, of which 74% are considered endemic [3]. This exceptional floristic diversity is threatened by accelerating economic development, mainly based on mining and metal processing activities [4]. Increasing human disturbances such as open-cast mining [5], fires [6], along with urbanisation and exotic species introductions [7], [8] have led to a reduction of 75% of the original vegetation cover since the arrival of man 3500 years ago [9], [10]. Establishment of conservation priorities in light of this environmental degradation requires sound knowledge on the distribution of plant biodiversity.

IUCN assessments form a major reference for the assessment of conservation status worldwide and have become a “powerful tool for conservation planning, management, monitoring and decision making” [11]. Local stakeholders use these assessments to establish lists of protected species on their territory, which take into account factors such as population size, growth rate, population fluctuations, habitat fragmentation and range size [12]. However, one of the difficulties of these assessments is that they are time consuming and require data that are not always available. Typically this involves obtaining information on contemporary population sizes or temporal changes in abundance, range or habitat quality. Because of this, Red Lists in tropical countries are known to be incomplete; therefore it’s difficult to estimate the number of truly threatened plant species at a global scale [13]. As a more immediate method of identifying species and areas of high conservation priority in New Caledonia, we have investigated the frequency and distribution of “narrow endemic species” (NES). These are species “that occur in one or a few small populations and hence are confined to a single domain or to a few localities” [14]. Our rationale is not to circumvent the Red-List process; rather it is to provide a first-pass floristic scale assessment of where the conservation issues are most likely to be concentrated and to target further threat assessment. Although widespread species may also be threatened, we focus on Narrow Endemic Species, as they are inherently vulnerable due to their limited distributions in a country with high levels of environmental degradation. This assessment of NES can then be used to target further more detailed conservation assessments on individual species and sites in the context of the IUCN red-list framework.

The use of herbarium records to obtain distributional data for rare or narrowly distributed species has already been identified as being useful in accelerating the establishment of conservation assessments [15] and for prioritizing conservation actions providing collection effort is taken into consideration [16]. We have adopted this approach for the flora of New Caledonia, focusing on distributional records of endemic angiosperm and gymnosperm species. This type of approach using available, but essentially *ad hoc* distributional data rather than *de novo* systematic sampling, has the limitation that under-sampled areas and species will obviously not register as important for conservation. This is pertinent in a country like New Caledonia in which some mountain ranges are difficult to access. In order to enhance our understanding of the ‘true’ distribution of narrow endemic species across New Caledonia, we have also used a modelling approach based on the distribution of ecological niches [17] to predict the distribution of the habitat of ecologically distinct groups of narrow endemic species.

Specifically, we tackle the following questions:

1. Using distributional data from herbarium specimens and taxonomic accounts:
  - How many species in the New Caledonian flora can be classified as narrow endemic species, and which species are these?
  - How many of these NES have IUCN conservation status?
  - For those species with IUCN conservation status, does their conservation status relate to their distributions, and if so, are there other currently ‘IUCN un-assessed’ species in the flora with similar distributions which warrant urgent conservation assessment?
  - Where are the Hotspots of Plant Narrow Endemism (HPNE) in New Caledonia, based on currently available data?
  - How well protected are NES and HPNE under existing conservation legislation?
  - To what extent do mining activities in New Caledonia represent a threat to NES and to HPNE?
  - Which are the NES and HPNE with least conservation protection and greatest threat from mining (e.g. the imminent ‘botanical car-crashes’)?
2. If the distribution of NES is modelled, does this provide additional insights into their occurrence and conservation needs?, specifically
  - Are there ecologically distinct groups within the community of NES in New Caledonia?
  - If so, what is the geographical distribution of the habitat that these groups occupy?
  - Are there areas in New Caledonia which are identified as ecologically suitable for NES which might shelter currently unrecorded communities of NES?

## Methods

### 2.1 Data Recording

We reviewed taxonomic descriptions and distributional data of all angiosperm and gymnosperm plant species (pteridophytes were discarded due to a shortage of distributional data and recent taxonomic revisions). This review used 25 volumes of “La Flore de Nouvelle-Calédonie” including 54 families of angiosperms and 5 families of gymnosperms and other taxonomic references (See Appendix S1). Distributional data were collected from the literature and from locality information on herbarium specimens from the IRD Nouméa (NOU) using the “Virot” database, Paris (P) and Zurich (Z) herbaria (the latter two were consulted through their websites (http://coldb.mnhn.fr/and http://www.zuerich-herbarien.ethz.ch)). The geographical coordinates (and their precision) of the numerous samples collected by H.S. MacKee (the most prolific New Caledonian plant collector) were obtained through a website dedicated to this collector (http://phanero.novcal.free.fr). Records also include unpublished personal communications on the description and distribution of species from different taxonomic specialists in charge of the revision of a family or a genus belonging to the New Caledonian flora: Pandanaceae (M. Callmander), Araliaceae and Myodocarpaceae (P.P. Lowry) and *Psychotria* Rubiaceae (L. Barrabé). The data collection ended in March 2011. Taxonomic groups that have not been subject to a recent revision, and which we considered the state of knowledge too limited were excluded. This involves the following families Clusiaceae, Eriocaulaceae, Gesneriaceae, Moraceae, Smilacaceae, Thymeliaceae, Xanthorrhoeaceae and the genera *Cyclophyllum* (Rubiaceae), *Desmodium* (Fabaceae), *Freycineta* (Pandanaceae), *Geniostoma* (Loganiaceae), *Piliocalyx* (Myrtaceae). In total, we included 2930 (86.9%) of the 3371 native species in the New Caledonian flora [3] in our assessment.

### 2.2 Assessment of Sampling Bias

Prior to further analyses, we checked for obvious bias in our data attributable to differential sampling effort. If some areas or taxa are systematically more heavily sampled than others this may lead to bias. An underlying problem in quantifying this bias is that overall sample effort of botanists over the last two hundred years is unknown, as is the ‘true’ distribution of narrow endemic species, and the degree to which there is an interaction effect between sampling practices and diversity. However, in New Caledonia, we do have the ability to assess this issue for a large proportion of the dataset, as almost a third of the records come from a single collector whose sampling effort is documented. To determine if the number of NES was correlated with sampling effort, we assessed whether the collection effort of H. S. MacKee was related to the number of NES that he found. For each cell of 2 km × 2 km we established the number of sampling trips that occurred and the number of NES found by this collector and tested for a relationship using Pearson’s correlation coefficient. We also tested how representative the sampling effort of MacKee is, in terms of the total number of samples of the New Caledonian flora in the Nouméa, Paris and Zurich herbaria. H. S. MacKee undertook more than 1600 collecting trips in New Caledonia. The correlation coefficient between sampling effort and number of NES per cell was very low (R = 0.117). The samples of MacKee represent 30.3% (n = 18565) of the total number of samples of the New Caledonian flora at the Nouméa herbarium, 27.4% (n = 14592) at the Paris herbarium and 9.9% (n = 1867) at the Zurich herbarium (a total of 27% of the records in the current study). In terms of species, 75.2% of the species in New Caledonia were collected at least once by MacKee and deposited at the Noumea herbarium. We conclude from this, that although some degree of recording bias in data of this type must be inevitable, that there is at least no markedly obvious bias in terms of sample effort and the recorded distribution of NES, for the component of our data in which this can reasonably be tested. Further exploration of the likelihood of some areas being under-recorded is undertaken in the species distribution model component of this paper.

### 2.3 Analysis of NES from Observed Distributional Data

We divided NES into three groups according to their range: narrow endemic species restricted to one location (NES 1 = sample positions are separated by less than 10 km), narrow endemics of two locations (NES 2 = not more than two sample positions separated by more than 10 km) and narrow endemic species restricted to 3 different locations (NES 3 = not more than three sample positions separated by more than 10 km). We recorded the number of species, which fell into each of these three categories. This definition of NES focuses on distributional discontinuities. There are a small number of cases where NES have a continuous distribution of records across a large area which become classified as a single site on account of the absence of a distributional discontinuity.

The IUCN status was recorded for each NES using the IUCN Red List website (www.iucnredlist.org). For the IUCN categories that were relevant to the dataset (extinct (EX), critically endangered (CR), endangered (EN), vulnerable (VU), lower risk (LR), near threatened (NT) and least concern (LC), we recorded which criterion was used for making the assessment (A – Declining populations, B – Geographic range size, and fragmentation, decline or fluctuations, C – Small population size, and fragmentation, D – Very small population or very restricted distribution, E – Quantitative analysis of extinction risk). We also recorded the number of species, which do not have an IUCN classification, and whether this was because they were ‘data deficient’ or whether they had simply not been assessed.

To relate IUCN status to distributional data we calculated the Area of Occupancy (AOO) and the Extent of Occurrence (EOO) of each NES, according to IUCN guidelines [12] using Mapinfo 10.5 when precise distributional data was available. These data allow us to compare distributional trends between NES with and without IUCN status. For each of the higher risk IUCN categories (CR, EN and VU) we correlated species with IUCN assessments against those NES 1, 2, & 3 species, which lack an IUCN assessment. This was to establish whether there are groups of un-assessed species, which show similar distributional patterns to known high-risk species. This was undertaken with a Pearson’s correlation coefficient.

To identify areas with high density of NES, we applied a grid of cells measuring 4 km^2^ (2×2 km) on the map of New Caledonia. For each type of narrow endemism (NES 1, NES 2, NES 3) the number of species occurring in a cell was identified. In this study, although we map all NES, we define a HPNE as where a total number >7 NES occur in a cell of 4 km^2^ (under this value the number of HPNE were to numerous to be detailed, they are however shown on our HPNE map). To relate these HPNEs to known geographical features, like named mountains or valleys, we recorded the total number of narrow endemic species and the number of NES 1 that occurred within the cells that make up these named features. In the case of isolated massifs this is relatively straightforward to do. However, in the continuous mountain systems in the south of New Caledonia, there is no consistent topographical rule set for placing hard boundaries. In these cases we subjectively allocated records at the boundary of interconnected mountains or valleys based on the best available data.

To establish the degree to which NES are protected by current conservation legislation, we used the lists of protected species in the South and the North provinces of New Caledonia [18], [19], which takes into consideration IUCN and also the Convention on International Trade in Endangered Species of Wild Fauna and Flora (CITES) status, and we also looked at the occurrence of these NES and of HPNE in the list of protected areas in New Caledonia [20]. We recorded which NES and HPNE were covered by species-based and area-based conservation protection. We also recorded for each species, the proportion of records which were within protected areas.

We then used maps of areas that have been impacted by mining activities [21] to establish which NES and HPNE were threatened by mining. We identified the proportion of records for a given species that are from a mining site, and which HPNE were on or adjacent to mining sites.

Based on the preceding analyses we compiled a list of the species and areas that are candidates for being the most threatened in New Caledonia. This was based on the number of NES not protected by local legislation, and the extent of threat from mining activities. In particular, we flag up NES and HPNE where conservation protection is low, and risk from mining activities is high.

### 2.4 Analysis of Environmental Data for Distributional Modelling of NES

We extracted topographic, climatic and substrate related environmental variables which are thought to be directly or indirectly related to the distribution of plant species in New Caledonia [22]. We included geology due to the hypothesised importance of substrate in plant communities [23] and in underpinning species radiations in New Caledonia [24].

For the topographic variables we used elevation based on the Shuttle Radar Topography Mission digital elevation model [25] with a resolution of 3 arc seconds (∼90 m). We also derived slope gradient from this high-resolution model using a function implemented in GRASS GIS [26]. With respect to climatic variables, for mean annual temperature we used global interpolated climate surfaces based on weather station data from 1950–2000 (“WorldClim”, [27]) at a resolution of 30 arc seconds (∼1 km). For precipitation we interpolated average mean monthly rainfall (over a period of 10 years from 1991–2000) from 121 weather stations using two different methods: (1) trivariate thin-plate splines based on x and y coordinates and elevation, implemented in GRASS GIS [28]; and (2) AURELHY (Analyse Utilisant le RELief pour l’HYdrométéorologie) – a method designed by Météo France [29] currently under development as a package for R [30]. The AURELHY method consists of 2 steps: first, the topography of the landscape is described at a range of scales by means of a Principal Component Analysis. Second, the weather station data are modelled using their x and y coordinates and the principal components as explanatory variables in a linear model whereby the residuals are krigged. We used the 90 m resolution digital elevation data for topography and derived the interpolation at a resolution of 1 km. Due to the linear nature of the model some overshoots were produced, and the predictions were truncated at 4900 mm in 0.2% of the cells. A jack-knifing procedure showed that the AURELHY method provided a closer fit to the weather station data than the thin-plate spline interpolation, and our AUREHLY interpolation also captured regional precipitation patterns better than the WorldClim and the downscaled Tropical Rainfall Measuring Mission 2B31 (atmospheric rainfall remotely sensed between 1997–2006; [31]) datasets. Consequently the AURELHY rainfall layer was used in all subsequent analyses. For geology we used a detailed and precise vector map (1∶50 000) [32] and assigned the original geology categories to 4 different groups that are thought to be of importance for plant distributions: ultramafic, volcano-sedimentary, limestone, and riverbanks/anthropic formations.

### 2.5 Developing a Distribution Model for Groups of Narrow Endemic Species

The data analysis consisted of two steps: (1) searching for ecological groups among the NES which have broadly similar habitat/environmental requirements, and (2) modelling the distribution of these groups.

### 2.6 Grouping Species Based on Environmental Similarity

As not all of these species have precise locality information suitable for linking to environmental data, we subsampled the 552 species from which we have precise record data and used these in the current study. For each species, we calculated the overall mean of environmental variables from the locations at which a species occurred (altitude, slope, total annual rainfall and mean annual temperature; all layers were resampled to a 1-km resolution as even though topography data exist at a finer resolution the specimen localities often do not). For the categorical variable geology we used the mode. Based on this information we derived a distance matrix between all species using Gower’s distance (symmetric distance measure designed to work with mixed data). Subsequently, we used three methods to explore whether there are any obvious discontinuities (ecological groups) amongst the species, and what the optimal number of groups are: (1) Partitioning around medroids (PAM) using (a) the Calinski-Harabasz index (based on ratio of within- to between-group similarity), and (b) optimum average silhouette; (2) hierarchical clustering (HC) (average links); (3) Metric (MDS) and Non-Metric Multidimensional Scaling (NMDS). All analyses were done in “R” [33] using the libraries ‘cluster’ [34], ‘vegan’ [35] and ‘fpc’ [36].

### 2.7 Modelling the Potential Distribution of Ecologically Distinct Groups of NES

Species Distribution Models (SDM) combine distributional data with environmental data from Geographical Information Systems (here altitude, slope gradient, rainfall, temperature and geology) to predict the distribution of suitable habitat for the target taxon. Typically SDM model single species but here we modelled the distribution of habitat for ecologically distinct groups of NES to identify the environmental space associated with high concentrations of NES. This approach was necessary as the vast majority of the NES under consideration do not have sufficient presence records to be modelled on their own. Modelling ecological groups of species instead of single species has been applied elsewhere [37], [38]. This approach makes the assumption that certain environmental conditions are associated with presence of a particular group of species, and by mapping the distribution of this environmental space in a given geographical area, one can hypothesise about the distribution of the set of species associated with this environmental space. The model output is thus obviously not representative of the distribution of any one individual species and instead just highlights areas of similar macro-environments to areas where high concentrations of NES have been recorded.

There is a risk that modelling groups instead of single species leads to over- or under-predictions of the extent of suitable habitat (depending on the shape of the environmental response curves of the individual species and the degree of overlap between them). Furthermore, particularly in the case of narrow ranging species, there is also the risk that relevant predictors have not been captured as the ranges may be narrow due to the species being dependent upon particular landscape features such as springs. The risk of unrepresentative predictions was reduced by working with ecologically distinct groups and by validating the model predictions on test data.

We removed duplicate records of individuals of the same species but not duplicate records of different species in any given grid cell. This means that the model results were weighted towards environmental conditions in cells with a high species richness of NES, thus HPNE. However, we also ran the model in a binary fashion, recording simple presence/absence of NES per grid square (e.g. with no weighting towards grid squares with >1 NES recorded). This approach was specifically designed to explore the sensitivity of the model to recording effort. Very similar results were obtained, indicating the robustness of the model to number of records per cell in the input data.

The model algorithm used was MaxEnt [39], an SDM method from presence-only records, which has consistently performed well in cross-model comparisons [40]. MaxEnt calculates the probability of occurrence (or more specifically the degree of habitat suitability) based on the density of the environmental covariates at the presence sites, and their density in the entire study area (background data). It searches for the solution that has maximum entropy (i.e. is closest to a null model whereby a species/species group has no environmental preferences), subject to the constraint that the means of the environmental covariates at sites that are predicted to have a high suitability are close the means across the observed presence locations [41]. We used the default setting for MaxEnt version 3.3.3 k (allowing for transformations of the covariates by enabling “auto-features” with the default thresholds for conversion, maximum number of background points = 10000; maximum number of iterations = 500; convergence threshold = 0.00001; fit regularization parameter = 1; default prevalence = 0.5), and we set aside 25% of the data as test data. Obviously these test data are only semi-independent as some will have been collected during the same sampling trips as the model training data and might therefore be subject to the same biases. Consequently, the test AUC values are likely to be somewhat higher than when based on a fully independent test dataset, and need to be interpreted with appropriate caution. The input data consisted of 86, 290 and 516 presence records for groups 1, 2 and 3 respectively (three ecologically distinct groups detected in the preceding analyses and described in the results section), and we modelled at a 1 km resolution.

### 2.8 Comparing HPNE from Distributional Data with the Distributions of NES Predicted from the Model

To identify areas that might shelter high densities of NES that weren’t identified based on distributional data alone, we compared the distribution of HPNE with high probabilities of occurrence for each map obtained with MaxEnt.

## Results

### 3.1 Analysis of Distributional Data

#### 3.1.1 Levels of narrow endemism in the New Caledonian flora and its relationship to IUCN classifications

Analysis of the New Caledonian flora showed that 309 species are recorded from only 1 location, 193 species from only 2 locations and 133 species from 3 locations. In total 21.7% of the examined component of the flora are classified as NES (635/2930 assessed species; Appendix S2). The vast majority of these species lack a formal IUCN conservation assessment (485/635 species). None are listed as data deficient; they have simply not been assessed at all. Of the 23.7% of NES which have a formal IUCN assessment:

- 0.5% (3 species) are considered as EX (1 of each NES 1, 2, 3)
- 4.7% (30 spp) as CR (6% (19) for NES 1, 5% (10) for NES 2, 1% (1) for NES 3)
- 7.4% (47 spp) as EN (7% (21) for NES 1, 9% (18) for NES 2, 6% (8) for NES 3)
- 8.6% (55 spp) as VU (8% (26) for NES 1, 7% (13) for NES 2, 12% (16) for NES 3)
- 2.4% (15 spp) as LR (0.6% (2) for NES 1, 3.1% (6) for NES 2, 5.3% (7) for NES 3)
- 0.2% (1 spp) as NT (0.3% (1) for NES 1)

Of the species classified as CR, 13 (43%) were classified using criteria which place a heavy weighting on distributional area (13 spp criterion B), for EN there are 43 (91%; 43 spp classed by criterion B) and VU there are 42 spp (76%; 26 spp B & 16 spp D2).

**Figure 1:**
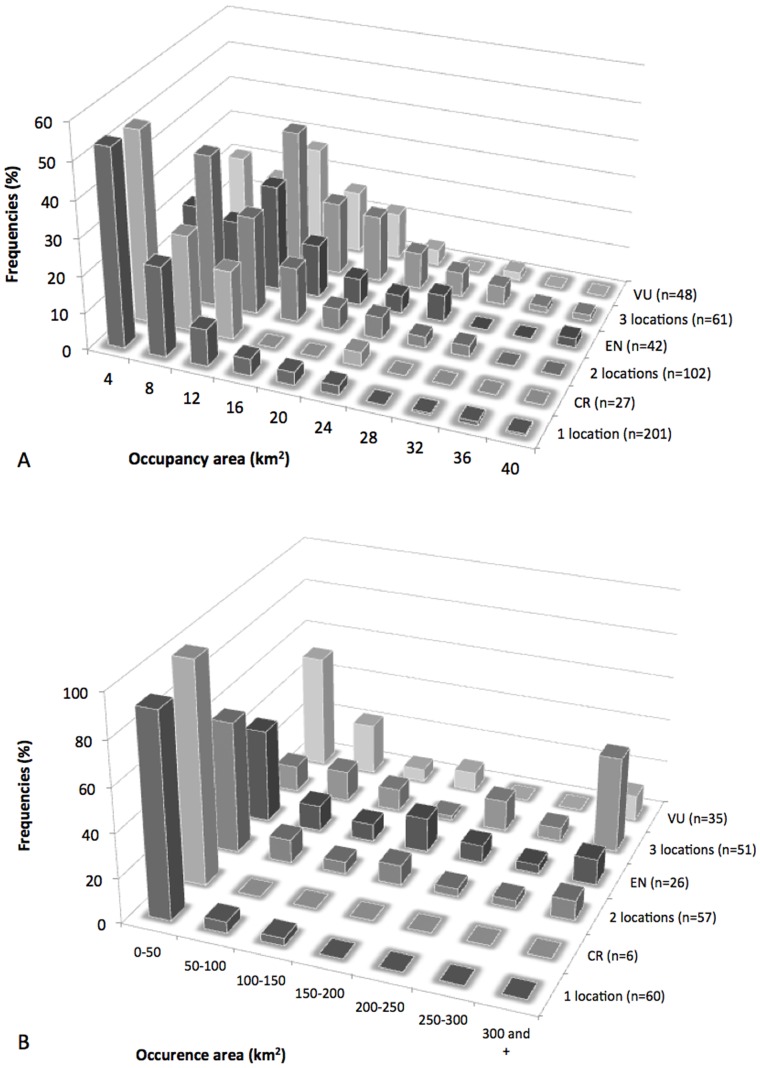
Frequency distributions of Area of Occupancy (A) and Extent of Occurrence (B) for New Caledonian Narrow Endemic Species. Comparing NES with IUCN status of CR, EN, VU, *or* are recorded from 1, 2 or 3 locations.

Considering the Area of Occupancy (Fig. 1), the frequency distribution of NES classified as CR match well the distributional data of the single site NES (R = 0.979, p<0.001). Likewise for the NES found at two sites (NES 2), the frequency distribution of their distributional data shows some concordance with those of NES with EN status (R = 0.641, p<0.05). For NES recorded from three localities (NES 3) no significant correlation is observed with IUCN status (CR: R = −0.141, p>0.05; EN: R = 0.576, p>0.05; VU, R = 0.577, p>0.05). Regarding the Extent of Occurrence (Fig. 1), discrimination is confounded due to the excess of data points in the smallest Extent of Occurrence category (50 km^2^), resulting in J-shaped frequency distributions. Thus NES 1 had trends similar to CR, EN and VU (respectively R = 0.998, p<0.001; R = 0.958, p<0.001; R = 0.919, p<0.01). Likewise NES 2 are similar to species classified as CR, EN and VU (respectively R = 0.991, p<0.001; R = 0.982, p<0.001; R = 0.948, p<0.01). For NES 3, no correlation is obvious with IUCN status (CR: R = −0.083, p>0.05; EN: R = −0.039, p>0.05; VU: R = 0.038, p>0.05). Exploration of different ‘bin’ sizes (categories on the X axis of the histogram), including splitting the smallest size category did not alter these findings.

#### 3.1.2 Hotspots of narrow endemic species

**Figure 2:**
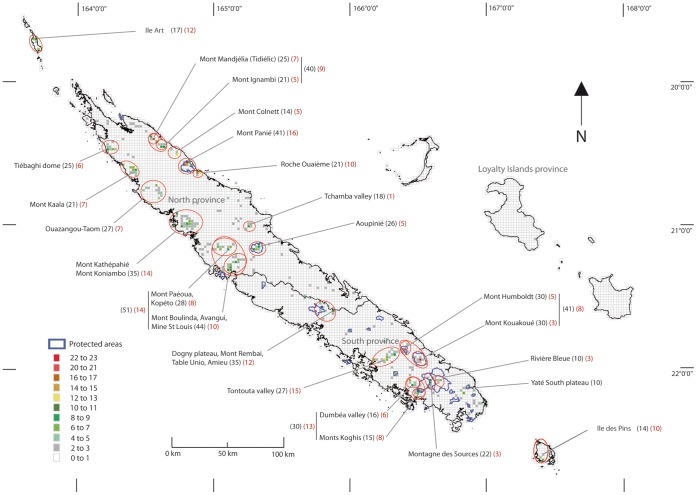
Map of “Hotspots of Plant Narrow Endemism” and protected areas in New Caledonia. Values given per cell are the total number of NES summing NES 1, 2 & 3. Red ellipses indicate collections of cells that fall within named geographical locations such as mountains or valleys. The total number of NES in the red ellipses is indicated in brackets beside the name of site, numbers in red refer to the total number of NES 1. The line in the middle of Grande Terre (the main island) is the separation between the north and the south provinces.

A map of HPNE (4 km^2^ with >7 NES) was produced (Fig. 2) and is summarised in tabular form in Table 1. More HPNE occur in the North province than in the South province (26 versus 10). Regarding the total number of NES (e.g. combining NES 1, 2 & 3), the highest numbers per 4 km^2^ cell are found on Mont Panié, Mont Kouakoué, Mont Humboldt, and Mont Mandjelia (all these HPNE, except the last one, are located in protected areas referring to category 1 b of the IUCN [42]). In the North, 14 HPNE are found on volcano-sedimentary substrates, 11 HPNE are found on ultramafic substrates and one is considered to be located on ultramafic and volcano-sedimentary substrate at the Tchamba valley. Apart from Mont Panié and Aoupinié, all the other HPNE in the North province are not protected. In the South province, most of the HPNE are found on ultramafic substrate except for the “Dogny plateau”. The Tontouta and the Dumbéa valley (ultramafic substrate) and the “Dogny plateau” (volcano-sedimentary substrate) are the only ones not protected by local legislation.

**Table 1:** Hotspots of narrow endemism (HPNE) in the New Caledonia flora where >7 NES are found per 2×2 km cell (see also Fig. 2).

HPNE name	Total number of NES	Number of NES 1 location	Number of NES 2 locations	Number of NES 3locations	Geographical position		Protected area	Miningimpacts	Substrate
					X	Y			
Mont Panié 1 (PN)	**22**	**7**	**7**	**8**	164°46′55″	20°33′54″	X		V
Mont Kouakoué (PS)	**22**	1	**13**	**8**	166°31′57″	21°57′35″	X		U
Mont Panié 2 (PN)	**21**	**9**	3	**9**	164°45′45″	20°34′59″	X		V
Mont Mandjelia (PN)	**20**	4	**10**	6	164°32′03	20°24′02″	-		V
Mont Humboldt (PS)	**20**	3	**12**	5	166°24′58″	21°53′16″	X		U
Roche Ouaième 1 (PN)	17	**8**	5	4	164°51′29″	20°38′17″	–		V
Aoupinié 1 (PN)	14	5	5	4	165°17′49″	21°10′56″	X		V
Mont Colnett (PN)	12	4	5	3	164°42′21″	20°30′37″	–		V
Roche Ouaième 2 (PN)	12	5	5	2	164°52′38″	20°38′17″	–		V
Tontouta valley (PS)	12	5	4	3	166°15′41″	21°57′38″	–	*	U
Tiébaghi dome (PN)	11	4	3	4	164°11′19″	20°28′10″	–	*	U
Tchamba valley (PN)	11	1	4	6	165°14′24″	21°01′10″	–		V/U
Monts Koghis (PS)	11	7	3	1	166°30′50″	22°10′36″	X		U/V
Montagne des Sources (PS)	11	2	5	4	166°36′38″	22°06′15″	X		U
Mont Koniambo 1 (PN)	10	4	1	5	164°44′23″	21°00′59″	–	*	U
Mont Boulinda 1 (PN)	10	3	6	1	165°06′13″	21°18′28″	–	*	U
Ile Art (Belep) (PN)	10	**9**	1	0	163°39′51″	19°41′10″	–		U
Avangui (PN)	10	2	5	3	165°05′03″	21°19′33″	–	*	U
Yaté South plateau (PS)	10	2	4	4	166°54′06″	22°09′25″	X		U
Mont Kaala (PN)	9	4	2	3	164°23′52″	20°36′58″	–	*	U
Mont Ignambi 1 (PN)	9	1	3	5	164°35′28″	20°27′19″	–		V
Kathépahié (PN)	9	4	5	0	164°42′08″	20°55′33″	–	*	U
Aoupinié 2 (PN)	9	1	3	5	165°18′59″	21°09′52″	X		V
Mine St Louis (PN)	9	2	3	4	165°06′12″	21°19′33″	–	*	U
Mont Boulinda 2 (PN)	9	3	5	1	165°08′32″	21°16′19″	–	*	U
Mont Koniambo 2 (PN)	9	5	1	3	164°46′42″	20°59′55″	–	*	U
Dogny (PS)	9	4	4	1	165°52′28″	21°37′03″	–		V
Montagne des Sources 2 (PS)	9	2	1	6	166°36′38″	22°07′20″	X		U
Mont Paéoua (PN)	8	4	3	1	165°05′06″	21°10'53″	–	*	U
Aoupinié 3 (PN)	8	4	3	1	165°18′59″	21°08′47″	X		V
Aoupinié 4 (PN)	8	2	4	2	165°16′40″	21°10′56″	X		V
Mont Panié 3 (PN)	8	4	1	3	164°46′55″	20°34′59″	X		V
Dumbea valley (PS)	8	2	5	1	166°28′30″	22°08′26″	–		U
Tontouta valley 2 (PS)	8	5	2	1	166°20′20″	21°55′27″	–	*	U
Mont Mandjélia 2 (PN)	8	1	5	2	164°30′55″	20°22′56″	–		V
Mont Ignambi 2 (PN)	8	0	3	4	164°36′37″	20°27′20″	–		V

The province of each “hotspot” is noted at the end of the name (PN: North province, PS: South province). An asterisk indicates that the site is potentially threatened by mining impacts. An “X” in the “Protected area” column indicates that the “hotspot” is protected. In the substrate column, “V” indicates a volcano-sedimentary substrate; “U” indicates an ultramafic substrate.

There is a small difference in terms of the importance of areas for the various classes of narrow endemism. The map and table summarise HPNE containing more than 7 NES when *all* NES are considered. When only NES 1 species are considered, the leading HPNE are Ile Art, Mont Panié, Roche Ouaième and Monts Koghis (of which Ile Art has a substantially lower ranking compared to when all NES are considered). For only NES 2, the most important sites are Mont Kouakoué, Mont Humboldt and Mont Mandjelia and for only NES 3 they are Mont Panié and Kouakoué (all high ranking sites in the *all* NES analysis).

When summing the number of NES per named geographical feature, Mont Boulinda/Avangui scores the highest richness in front of Mont Panié, Mont Kouakoué/Mont Humboldt and Mont Mandjelia/Mont Ignambi. In addition, the cumulative records for the Mont Koniambo/Mont Kathépahié massif and the Dogny/Remabai/Amieu/Unio area also contain >30 NES species. For NES 1 species only, Mt Panié is the top ranking site, followed by the Tontouta valley, the Mont Boulinda/Kopéto/Mont Paéoua, the Mont Koniambo/Mont Kathépahié massif, the Dogny/Remabai/Amieu/Unio area, and Ile Art (Fig. 2). No HPNE were found in the Loyalty Islands.

#### 3.1.3 Conservation protection of NES

An evaluation of the conservation status of NES species showed that in the South province 126 species of angiosperm and gymnosperm are on the list of protected species (and listed at the species level), and 105 of these (83%) are NES (57 NES 1, 30 NES 2 and 18 NES 3). In the North province, 268 species are listed at the species level, and 148 of these (55%) are NES (67 NES 1, 49 NES 2 and 32 NES 3). Overall, 32% (206) of NES have some level of species-based conservation protection. In terms of protected areas, 74% (473) of species have no records from protected areas, and a further 7% (46) of species have >50% of their records outside protected areas. In total, 306 NES have no protection, either under species-based, or area-based measures.

Of the 36 HPNE identified in this study, only 13 are in protected areas. These protected HPNE are mainly located on volcanic sedimentary substrates (8) and ultramafic substrates (5).

#### 3.1.4 Mining threats to NES

In terms of the impact of mining activities, nearly 1/3 of NES (30%) have at least one record located in an impacted area (24.3% (75) for NES 1, 28.9% (56) for NES 2, 44% (59) for NES 3), and 55 species (9%) have all of their records in a mining impacted area. For only the species on ultramafic substrates, this value exceeds 50% of the species (190) with at least one impacted site (41% for NES 1, 50% for NES 2, 73% for NES 3). A third of all HPNE (12) are impacted by mining activities. These areas are not protected by local legislation and they are all located on ultramafic substrate (see Table 1).

Appendix S2 lists the NES, unprotected by local legislation, from which all of their recorded locations are in areas impacted by mining (37 NES in total (6%), 27 NES 1 (9%), 6 NES 2 (3%), 4 NES 3 (3%)), or where at least 50% of their recorded locations are impacted by mining (38 NES in total (6%), 9 NES 1 (3%), 12 NES 2 (6%), 17 NES 3 (13%)). These 75 species are highlighted as being of the highest conservation concern.

### 3.2 Modelling the Distribution of NES

#### 3.2.1 Evidence for ecological distinct groups of NES

The HC and NMDS analyses showed that there were three distinct groups of NES, separated mainly by their occupied geological substrates: ultramafic, volcano-sedimentary and limestone or riverbanks/anthropic formations. We discarded the results from the PAM method which separated the data into 4–6 groups, because no isolated clusters were found, and visual inspection of the dissimilarities (hierarchical cluster plot and NMDS) did not support the notion that there were 4–6 clusters (only 3 clusters were visually distinguishable). No further subgroups were apparent when further clustering/ordination analyses were carried out separately for each of the identified groups. Topography and climate variables (inter-correlated) seem to act more as continuous gradients with both ultramafic and volcano-sedimentary NES covering a wide range of these.

**Figure 3:**
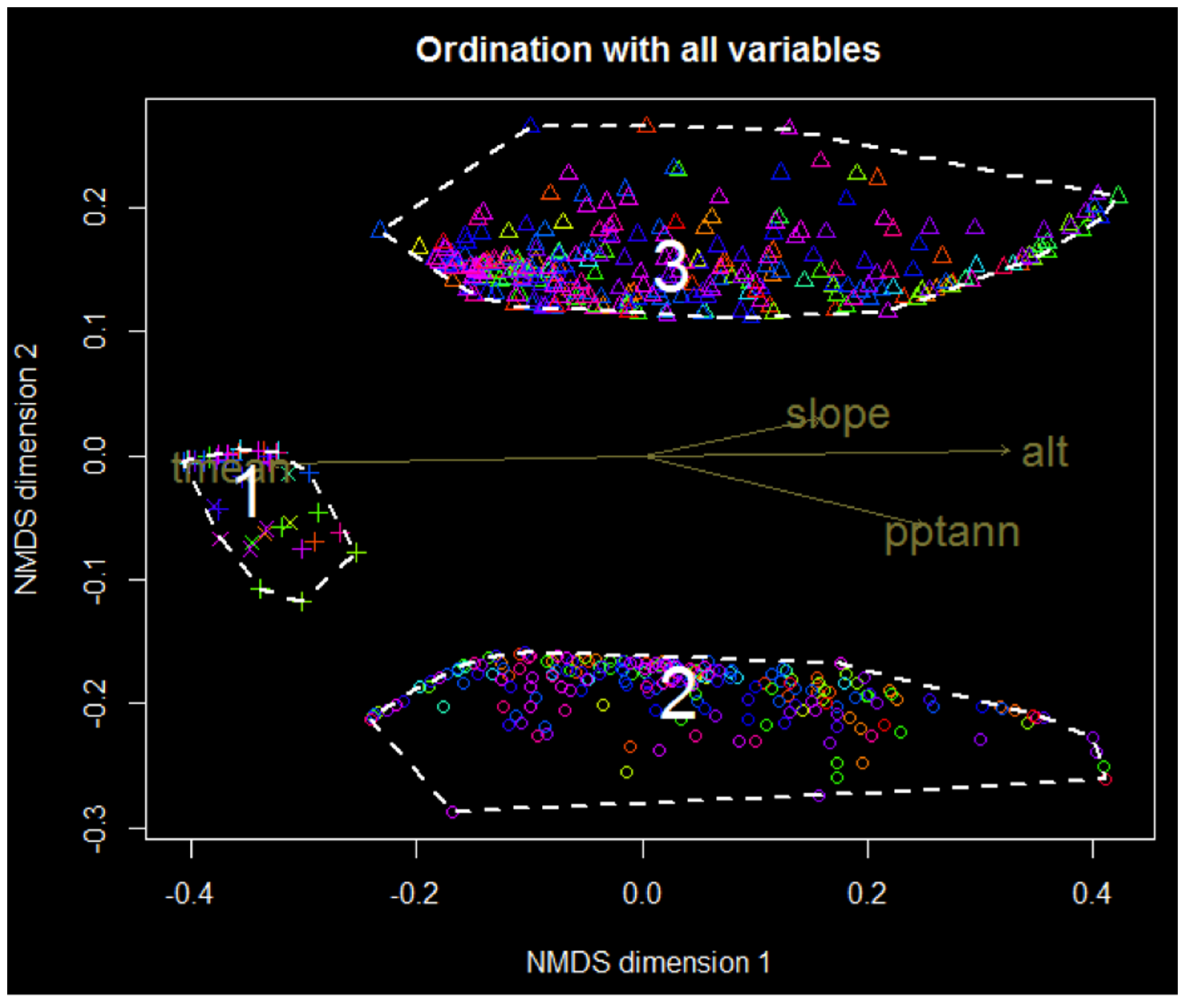
Groups of NES according to environmental variables. (pptann : total rainfall per year, tmean: mean temperatures, alt: altitude). Each point is a unique NES. Symbols represent the geological substrate of the NES (o: volcano-sedimentary, Δ: ultramafic, +: anthropic formations and riverbanks, x: limestone). Points are coloured according to taxonomic families.

The three groups are the following (Fig. 3):

1. A small group (n = 43) which occur at low altitudes ( = higher mean temperature, and lower annual rainfall) either on limestone or anthropic/riverbanks substrates (named G1)
2. A large group of species (n = 225) which occur on volcano sedimentary substrates. This species group covers a wide range of altitudes, temperatures, rainfall, and slope steepness (G2)
3. A large group of species (n = 284) occurring on ultramafic substrates, along an equally wide range of altitudes, temperatures, rainfall, slopes as Group 2. (G3)

#### 3.2.2 Modelling of the potential distribution of groups of NES

**Figure 4:**
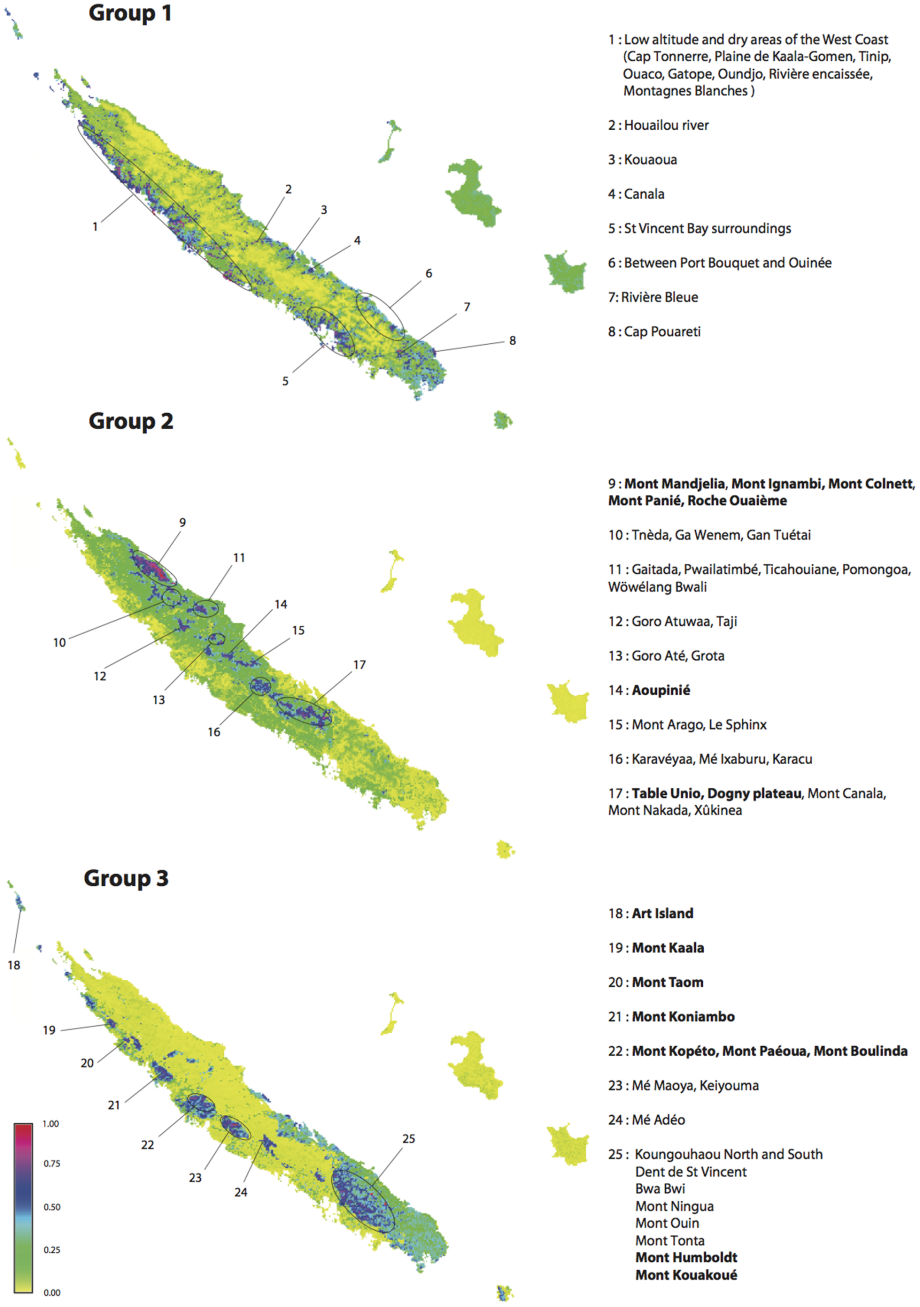
Predicted distributions of the three groups of NES. For each group, the map of potential distribution (colour according to probability of the habitat being suitable), and the name of location presenting the highest probabilities for a suitable habitat are detailed (Areas already identified as HPNE are in bold).

The potential distributions of each group and results of MaxEnt runs are summarized in Fig. 4.

Group 1: It was possible to model this group with reasonably good predictive power for the test data (training AUC = 0.89, test AUC = 0.83). Substrate was the most important variable (contributing 66%; model performance declining by 64% when this covariate is permuted). The main substrates concerned are anthropogenic/riverbanks and limestone. Altitude was of moderate importance (17%). Other variables have much less explanatory power in the model (<10%). It should however be noted that altitude and temperature were correlated (Pearson’s R = −0.95) and that the climatic and topographic variables are also partly linked to the substrate (e.g. anthropogenic/riverbanks and limestone substrates occur at low altitudes where temperatures are higher).

These correlations amongst the covariates mean that care has to be taken when interpreting the importance of individual covariates (they can replace each other, and explanatory power of a given covariate may have wrongly been attributed to a different covariate).

As multi-colinearity can also affect marginal response curves we assessed the effect of each environmental variable on the prediction based on a model created using only the corresponding variable. This showed that habitat suitability decreased with altitude and slope, increased with temperature and is bimodal along rainfall gradient (peaks around 1000 and 4900 mm rainfall).

Group 2: This model also showed good predictive power (training AUC = 0.87, test AUC = 0.84). Substrate was again the most important variable (contributing 42%; model performance decreasing by 70% when this covariate is permuted). The main substrate of this group was volcano-sedimentary. Mean temperature and altitude were also important (27% and 24% respectively). Contributions of other variables were below 10%. Habitat suitability increased with altitude and rainfall and decreased with temperature.

Group 3: This model showed the highest predictive power (training AUC = 0.89, test AUC = 0.87). Geology had a high contribution (contributing 74%; model performance declining by 70% when this covariate is permuted). The main substrate was ultramafic rocks. Altitude contributed 17% and all other variables less than 10%. Habitat suitability increased with altitude, rainfall and slope, and decreased with temperature.

#### 3.2.3 Comparing current HPNE and groups predicted distributions

Areas of a high probability of sheltering NES for a given group are shown in Fig. 2. For each location, an asterisk indicates that it has already been identified as a HPNE based on observed distributional data. For group 1, Rivière Bleue has previously been identified as rich in NES (Fig. 2), but the model also predicted additional sites with a high probability (>75%) of occurrence of NES (e.g. Low altitude and dry areas of the West Coast (Cap Tonnerre/Plaine de Kaala-Gomen/Tinip/Ouaco/Gatope/Oundjo/Rivière encaisée/Montagnes Blanches and surrounding area of St Vincent Bay) and of the East Coast (Houailou river; Kouaoua; Canala; between Port Bouquet and Ouinée, and the Cap Pouareti)). For group 2, several sites were identified as hotspots from the distributional data *and* with a high probability of occurrence for NES (Mont Panié and neighbouring mountains, Aoupinié, and the Dogny plateau and surrounding area), with additional sites predicted by the model (e.g. Tnèda/Ga Wenem/Gan Tuétai/Gaitada/Pwailatimbé/Ticahouiane/Pomongoa/Wöwélang Bwali; Goro Atuwaa/Taji; Goro Até/Grota; Mont Arago/LeSphinx; Karavéyaa/Mé Ixaburu/Karacu; Mont Canala/Mont Nakada/Xûkinea). Finally for group 3, Ile Art, Mont Kaala, Mont Taom, Mont Koniambo, Mont Kopéto/Mont Paéoua/Mont Boulinda, Mont Humboldt and Mont Kouakoué were identified as hotspots from the distributional data and with a high probability of occurrence for NES, with additional sites predicted by the model (e.g. Mé Maoya-Keiyouma, Mé Adéo, Koungouhaou North and South/Dent de St Vincent/Bwa Bwi/Mont Ningua/Mont Ouin/Mont Tonta).

## Discussion

### 4.1 Diversity of NES and their IUCN Classification

In the current paper we present the first formal floristic-scale assessment of the extent of localised endemism in New Caledonian plants. We show that a high proportion of the flora can be classified as ‘Narrow Endemic Species’ based on the available data. In total 22% of the assessed species in the flora have a maximum of three distributional points (defined as a record, or a cluster of records separated by >10 km from other records). It is of course possible that further survey work will identify additional populations of these species and we fully accept that some of these species will turn out to be more common. Nevertheless, this represents the best currently available dataset on the distribution of narrow endemic species in the New Caledonian flora. It suggests that a high proportion of the flora may have an inherent vulnerability to environmental perturbations due to having extremely restricted distributions and hence being susceptible to single catastrophic events triggering extinction.

In the context of the large number of narrow endemic species in the New Caledonian flora, the Red-listing of New Caledonian species is clearly in need of updating. The first major assessment of the conservation status of New Caledonian vascular plants was undertaken in 1994. At that time 392 species were identified as either LR, VU, EN or CR. The most recent version of the IUCN Red List (2011.1) included 44 species of gymnosperm and 313 species of angiosperm from New Caledonia (www.iucnredlist.org). Of the 635 New Caledonian NES identified in the current study, 76% have not been subjected to an IUCN conservation assessment (Fig. 3); and of the 150 NES which have been assessed, 77 have been given the conservation status of Endangered or Critically Endangered (the remaining species are classified as either extinct, vulnerable, lower risk and near threatened). The obvious implication of this is that many of the un-assessed NES may also warrant formal IUCN conservation status. The IUCN guidelines [12] do not allow listing of a species as Endangered or Critically Endangered based purely on distributional data, and we concur that (a) distributional data per se are only a proxy measure for threat, and (b) that our distributional data are imperfect.

Nevertheless, based on the precautionary principle, it is worth noting the strong statistical correlation in the frequency distributions of Areas of Occupancy between un-assessed NES 1 and species assessed as Critically Endangered; and likewise, the statistically significant (albeit weaker) correlation between NES 2 and species assessed as Endangered. We suggest this information is used in two ways. The first and obvious point is to flag up these NES as being in urgent need of formal IUCN assessment and further distributional surveys.

The second point, is that the known distributions of NES species can be used to underpin rapid threat assessment based on best available knowledge, by providing immediate information as to which species (or proportion of known records of a species) are likely to be lost if a habitat destruction occurs at a given location.

### 4.2 Hotspots of Narrow Endemic Species

This study has identified distinct Hotspots of Plant Narrow Endemism (HPNEs; Fig. 2; Table 1). This approach and the terminology of HPNEs has been used elsewhere (e.g. “Hotspot of global and local rarity” in California, [43]; “Hotspot of narrow endemism” in Madagascar, P.P. Lowry pers. com. 2011). In part, these concentrations of records yielding high NES richness in a given location may be attributable to sampling artefacts [44]. This is inevitable. However, our tests on a major subset of the data (collections by MacKee) suggest that collection effort is not the major driving factor in the patterns we observe. Beyond this, establishing what proportion of the signal is attributable to data bias is problematic. The *ad hoc* nature of the sampling over a 200 year period, and the unknown extent to which diversity has driven sampling effort (e.g. researchers targeting interesting areas), versus diversity patterns being attributable to sampling effort prevents robust internal corrections for data bias. We instead make the simple observation that we have identified the sites where most narrow endemic species have been recorded. This information on its own reflects useful knowledge for conservation planning in the absence of a perfect understanding of diversity and distributions.

The HPNE identified in this study have in certain cases already been identified by the scientific community as areas containing high numbers of endemic species. This is true for high altitude communities on volcano-sedimentary soils on Mont Panié [45] and Mont Mandjelia (Fig. 2; Table 1). Likewise, HPNEs on ultramafic substrates already identified and protected include high altitude communities on geric ferralsols on Mont Humboldt and Mont Kouakoué [46], [47].

The interpretation of HPNE based on a 4 km^2^ grid approach has both strengths and limitations. Its strength is an objective standardised area from which diversity patterns can be compared. However, a general problem with grid-based approaches is that the starting point for a grid can influence whether an individual cell scores highly, or has its species distributed either side of the boundary of adjacent cells. In addition, in situations where species richness is equal and evenly distributed, an open terrain is likely to lead to a more diffuse spread of records among cells compared to the situation where difficult terrain enforces concentration of collection effort into a narrow geographical space.

Nevertheless – the results of the top-ranking sites based on the grid-based approach, are broadly congruent with the hotspots identified from summing the records for a given massif or valley. Thus, when aggregating records into named geographical features, Mont Panié, Mont Humboldt and Mont Kouakoué remain among the highest ranking sites, and Mont Mandjelia is above the HPNE richness mid-point, particularly when considered as part of a contiguous range with the neighbouring Mount Ignambi (Fig. 2). In addition, sites such as the Koniambo/Kathépathié massif and its surrounding area, Mont Boulinda and its surrounding area, the Dogny Plateau/Mont Rembai/Table Unio/Amieu complex, the Tontouta Valley and Ouazangou-Taom also contain large numbers of NES. This same set of sites also scores highly when only NES 1 species are considered (Mont Boulinda/Kopéto/Mont Paéoua, the Mont Koniambo/Mont Kathépahié massif, the Dogny/Remabai/Amieu/Unio area). One additional site which scores highly for single site endemics is Ile Art. This island is interesting, as though its total number of NES is modest, a large proportion of these are NES 1 (12/17). This may be attributable to the small isolated nature of the island (a similar NES 1: NES total ratio is found on the similarly isolated Ile des Pins, 10/14).

### 4.3 Conservation Implications

Our review of the conservation status of narrow endemic species shows that about 50% of all NES lack conservation protection in the form of either species-based listings, or site-based protection. Furthermore, 64% of the HPNE lack conservation protection. Fully accepting that our data represent an imperfect understanding of the distribution of these NES, we consider this information useful for conservation planning based on ‘best available evidence’.

An important threat to NES in New Caledonia comes from mining activities. These threats are predictable (based on the distribution of metal-rich soils, and site accessibility) and deterministic (e.g. mining activities are planned). The impact of mining activities in New Caledonia on plant biodiversity has already been discussed by numerous authors [4], [5], [48], [49], [50]. These impacts are expected to increase with the planned rise in nickel production of 60,000 to 200,000 tonnes per year between 2013 and 2015 [4]. Although improvement in both mining and re-vegetation techniques have occurred [51], [52], it is inevitable that this scale of mining activity will have further major impacts on plant biodiversity in New Caledonia.

The greater the proportion of a species range that occurs in a mine-impacted area, and the lower the protection status of that species, the greater the conservation problem. Examples of species facing immediate threats include rare species with very limited distributions in the vicinity of active mines such as *Alyxia veillonii* (Apocynaceae) from the Tontouta valley, *Scaevola barrierei* (Goodeniaceae) from Mont Kopéto and *Litsea racemiflora* (Lauraceae) from the Tiebaghi dome (Appendix S2). These species need active conservation intervention to avoid extinction in near future. In terms of hotspots of narrow endemic species which are close to existing mines, there are a number of sites with hypermagnesian soils (magnesic cambisols) on serpentinites, such as the Tontouta valley and Avangui [53]. However, the threat to these areas is less immediate as the low nickel concentrations in this substrate reduce the likelihood of mining (although the sites are still subject to fires and invasive species). Some of the most critical sites are the west coast ultramafic mountains on high-nickel concentration gerric ferralsols which are actively mined such as the Tiebaghi dome, Mont Kaala, Ouazangou-Taom Mountains, Mont Koniambo, Mont Kopéto, Mont Paéoua and Mont Boulinda. These sites experience major impacts from mining activities and are not protected by local legislation.

Mining is a well publicised and highly visible threat to biodiversity on New Caledonia, and New Caledonia contains between 20 - 30% of the world’s nickel resources [4]. However, impacts from fire also represent a major conservation problem. In 2005, a severe fire in the close vicinity of one of the most tightly protected areas on New Caledonia (Montagne des Sources) provided a clear illustration of this problem and approximately 4000 ha of land were burnt. Thus the non-protected HPNE on volcano-sedimentary substrates like the Mont Colnett, Mont Ignambi [43], [54], [55], [56], the Roche Ouaième [57], and the Dogny plateau [56] which are not threatened by mining, are still of concern due to potential fire damage. On a positive note, the site with the highest diversity of NES (Mont Panié) is protected by the North province legislation and protected from fires to some extent by the lack of forestry roads and the extent of its evergreen forests.

Other threats to biodiversity on New Caledonia include logging (as observed on HPNEs such as Mont Mandjelia) and the spread of exotic plant species such as *Pinus caribaea*.

### 4.4 Insights from Distributional Modelling of Groups of Narrow Endemic Species

In this study, we modelled the distribution of groups of NES. It is important to stress from the outset that this approach simply searches for environmental conditions associated with high concentrations of NES, then extrapolates this association across New Caledonia. This highlights areas with similar environmental conditions which may consequently be suitable for harbouring high concentrations of NES. The modelling approach is of course unsuitable for predicting the occurrence of any individual species.

Three distinct groups of NES have been detected, separated mainly by their substrates. NES of ultramafic soils and volcano-sedimentary substrates are the most numerous. Although the habitat of volcano-sedimentary substrates has the largest extent in New Caledonia, this study shows slightly higher numbers of endemic species on ultramafic substrates, despite it covering only 1/3 of the territory. Adaptation to patchily distributed ultramafic substrates is thought to be a driver of speciation in New Caledonia [58], [59]. Similar conclusions have been reached from elsewhere in world, such as California and Cuba [60].

Considering the groups individually, Group 1 is located mainly on limestone/river banks/anthropic substrates and has relatively few species and this habitat type is scarce throughout the main Island. In addition to Rivière Bleue, which is rich in NES according to the distributional data, several other sites are modelled as having a high probability of containing NES (Fig. 4). However, these areas have encountered severe degradation due to human activities. Dry forests previously found in these areas have been mainly replaced by pasture for cattle, and the current remaining patches are thought to represent only 2% of their initial area [61]. Fires also frequently impact these areas. Thus although the model predicts a high probability of occurrence of NES based on environmental data in some sites, anthropic disturbance has undoubtedly reduced the amount of currently suitable habitat and extinctions are likely to have occurred.

The numerous species of Group 2 are located mainly on volcano-sedimentary rocks and habitat suitability increases with altitude and rainfall and decreases with temperatures. This indicates the importance of high altitude plant communities in New Caledonia which matches studies from other areas of the word [62], [63]. The Mont Panié mountain range - up to Mont Mandjelia – is the region with the highest and most clustered area of habitat suitability and is well known (and visited) by the botanic community for its plant richness. The Aoupinié massif and the Dogny/Mont Rembai/Table Unio complex are also areas where many samples of NES have been recorded and are modelled as having high habitat suitability. Areas which indicate the presence of high habitat suitability, which do not have large numbers of NES recorded, include Tnèda/Ga Wenem/Gan Tuétai; Gaitada/Pwailatimbé /Ticahouiane/Pomongoa/Wöwélang Bwali; Goro Atuwaa/Taji; Goro Até/Grota; Mont Arago/LeSphinx; Karavéyaa/Mé Ixaburu/Karacu and Mont Canala/Mont Nakada/Xûkinea. Further sampling in all these areas would be useful as they are likely to be under-recorded due to inaccessibility.

For group 3, found on ultramafic substrates, nearly all the areas where this substrate occurs are identified as suitable habitat for narrow endemism. The one exception to this is an ultramafic region in the south of New Caledonia with low probabilities to shelter NES. This can be explained by the large continuum of ultramafic substrates and low altitude in that area which may result in individual species being more widespread (e.g. not narrowly endemic) compared to those in more topographically heterogenous areas. In group 3, the NES habitat suitability increased with altitude, rainfall and slope and decreased with temperatures. Most of the areas of highly suitable habitat for NES in G3 have already been identified as important based on distributional data (Fig. 2 and 4). However, some additional locations are identified here. This is particularly the case of the Mé Maoya/Keiyouma massif, which is identified in the model of having a high probability for the presence of NES. This site is not currently impacted by mining activities, however, an increased of sampling effort to establish its richness in NES would be timely based on the results presented here. This is also the case of Mé Adéo/Dent de St Vincent/Bwa Bwi/Mont Ningua which appear under sampled according to their high suitability for NES, but the currently low number of species recorded. In contrast, Koungouhaou North and South is relatively easily accessible and in general well sampled, but fires may have caused the apparent discrepancy between its suitability for NES as predicted by the model, compared to the relatively low number of NES recorded. Likewise, Mt Ouin and Mont Tonta are predicted as having a high probability of occurrence of NES, but frequent fires are known to have led to habitat destruction at this site, and hence may have reduced the observed diversity of NES.

Appendix S3 provides a list of the main sites with a high likelihood of containing NES that were not recorded as HPNE based on the distributional data. This is accompanied by an informal indication of whether we consider this as most likely to be due to anthropic degradation leading to local extinction, versus them being under-recorded due to inaccessibility.

## Conclusions

This study provides an assessment of the extent of narrow species endemism in the New Caledonia flora, and identifies a set of species and locations of potential conservation importance. These data represent a baseline for more detailed studies of conservation status according to IUCN guidelines, and an immediate information resource until these more detailed studies have been undertaken. The study also indicates the distribution of suitable habitat for NES in New Caledonia, and hence can be used to help prioritise areas needing further field work and ultimately whose conservation status may need re-assessing. Modelling the distribution of habitat types suitable for narrow endemism also provides a useful baseline for evolutionary/speciation studies aiming to understanding the environmental correlates and drivers of species diversity.

Having this first-pass assessment of the distribution of NES in New Caledonia is important given the project rise in nickel production in the next 2 years. Nickel mining accounts for 95% of export income for New Caledonia, and effective exploitation of this resource is central to the island’s economy. Greater clarity on the distribution of plant biodiversity enhances understanding of the likely impacts of mining activities, and facilitates the development of the most effective strategies for reducing biodiversity loss.

## Supporting Information

Appendix S1 **References used for the revision of the whole new Caledonian flora.**
(PDF)

Appendix S2 **List of narrow endemic species (1, 2 and 3 locations).**
(PDF)

Appendix S3 **Sites with a high likelihood of containing NES that were not identified as HPNE from the distributional records alone.**
(PDF)
