# Supplementary material for: Conservation Priorities in a Biodiversity Hotspot: Analysis of Narrow Endemic Plant Species in New Caledonia
Source: PLoS One. 2013 Sep 18;8(9):e73371. doi: 10.1371/journal.pone.0073371 (PMC3776834; doi:10.1371/journal.pone.0073371)
Supplement: Appendix S1 — References used for the revision of the whole new Caledonian flora. (PDF) [file pone.0073371.s001.pdf]

Appendix 1:

References used for the revision of the whole new Caledonian flora.

Achille F (2006) *Tinadendron*, nouveau genre de Rubiaceae, Guettardeae de Mélanésie orientale. Adansonia 28(1): 167-180.

Anderson C (2011) Revision of *Ryssopterys* and transfer to *Stigmaphyllon* (Malpighiaceae). Blumea 56: 73 -104. doi: 10.3767/000651911X573444.

Armstrong JA (2002) *Zieria* (Rutaceae): a systematic and evolutionary study. Aust Syst Bot 15: 277-463.

Aubréville A, Leroy J-F, MacKee HS, Morat P (1967-) Flore de la Nouvelle-Calédonie et Dépendances. Muséum National d'Histoire Naturelle, Paris.

Barrabé L, Mouly A, Munzinger J (2011) Deux espèces nouvelles de *Thiollierea* (Rubiaceae) restreintes aux sols hypermagnésiens du massif du Boulinda (Nouvelle Calédonie). Adansonia 33: 135-148.

Bradford J, Jaffré T (2004) Plant species microendemism and conservation of montane maquis in New Caledonia: two new species of *Pancheria* (Cunoniaceae) from the Roche Ouaïème. Biodivers Conserv 13(12): 2253-2274. doi: 10.1023/B:BIOC.0000047901.33761.3c.

Dawson JW (*in prep*) Myrtacees. Myrtoidees. Eugenia.

Hartley TG (1982) A Revision of the Genus *Sarcomelicope* (Rutaceae). Aust J Bot: 30, 359-372. doi: 10.1071/BT9820359.

Hartley TG (1985) A Revision of the Genus *Medicosma* (Rutaceae). Aust J Bot: 33, 27-64. doi: 10.1071/BT9850027.

Hartley, TG (2003) *Neoschmidia*, a new genus of Rutaceae from New Caledonia. Adansonia 25(1): 7-12.

Hartley TG, Mabberley DJ (2003) The identity of *Picrella* Baill. (Rutaceae) with a revision of the genus. Adansonia 25(2): 251-259.

Hopkins HCF, Pillon Y, Bradford JC (2009) The endemic genus *Pancheria* (Cunoniaceae) in New Caledonia: notes on morphology and the description of three new species. Kew Bulletin 64(3): 429-446. doi: 10.1007/s12225-009-9137-z.

Hopkins, HCF (2007). *Geissois bradfordii*, a new species of Cunoniaceae from New Caledonia. Kew Bulletin 62: 275-280.

Hopkins, HCF, Fogliani B, Pillon Y (2007) Four new species in the endemic genus *Codia* (Cunoniaceae) from New Caledonia. Kew Bulletin 62: 259-273.

Jaffré T, Munzinger J, Lowry PP (2010) Threats to the conifer species found on New Caledonia's ultramafic massifs and proposals for urgently needed measures to improve their protection. Biodivers Conserv 19(5): 1485-1502. doi: 10.1007/s10531-010-9780-6.

Jaffré T, Fambart J (2002) Quatre nouvelles espèces de *Soulamea* (Simaroubaceae) de Nouvelle-Calédonie. Adansonia 24(2): 159-168.

Kostermans AJGH (1977) Un nouveau *Litsea* (Lauraceae) de Nouvelle-Calédonie. Adansonia *2*(17).

de Laubenfels DJ (2003) A new species of *Podocarpus* from the maquis of New Caledonia. New Zeal J Bot 41: 715-718.

de Laubenfels, DJ (2005) Statut du *Podocarpus* de l'Île des Pins (Nouvelle-Calédonie). Adansonia 27(2): 151-153.

Lowry PP, Plunkett GM, Raquet V (2004) Inclusion of the endemic New Caledonian genus *Pseudosciadium* in *Delarbrea* (Apiales, Myodocarpaceae). Adansonia 26(2): 251-256.

Mcpherson G, Lowry PP (2004) *Hooglandia*, A newly discovered genus of Cunoniaceae from New Caledonia. Annals of the Missouri Botanic Garden 91(2): 260-265.

Morat P, Chalopin M (2003) Quatre nouvelles espèces d'*Acropogon* (Malvaceae: Sterculieae) endémiques de la Nouvelle-Calédonie. Adansonia 25(2): 191-203.

Morat P, Chalopin M (2007) Contribution à l'étude des Malvaceae, Sterculieae de la Nouvelle-Calédonie : nouvelles espèces dans le genre *Acropogon* Schltr. Adansonia 29(1): 93-104.

Morat P, Chalopin M (2005) Quatre autres nouvelles espèces d'Acropogon Schltr. (Malvaceae, Sterculieae) endémiques de Nouvelle-Calédonie. Adansonia 27(2): 255-266.

Mouly A, Hoang N (2007) Une nouvelle espèce d'*Ixora* (Rubiaceae) cauliflore de Nouvelle-Calédonie. Adansonia 29(1): 123-128.

Mouly A, Pisivin C (2007) Rare and threatened new endemic *Ixora* (Rubiaceae) from New Caledonia. Nord J Bot 25: 14-19. doi: 10.1111/j.2008.0107-055X.00037.x.

Munzinger J (2001) Two new species of *Agatea* (Violaceae) endemic to New Caledonia, with some taxonomic notes and a key to New Caledonian species. Bot J Linn Soc 137: 91-97. doi: 10.1006/bojl.2001.0457.

Munzinger J, McPherson G, Lowry PP (2008) A second species in the endemic New Caledonian genus *Gastrolepis* (Stemonuraceae) and its implications for the conservation status of high-altitude maquis vegetation: coherent application of the IUCN Red List criteria is urgently needed in New Caledonia. Bot J Linn Soc 157(4): 775-783. doi: 10.1111/j.1095-8339.2008.00804.x.

Munzinger J, Swenson U (2009) Three new species of *Planchonella* Pierre (Sapotaceae) with a dichotomous and an online key to the genus in New Caledonia. Adansonia 31(1): 175-189.

Nielsen IC (2004) A new inland species of *Canavalia* (Leguminosae, Papilionoideae) from New Caledonia. Adansonia 26(2): 149-151.

Nielsen IC, Labat J-N, Munzinger J (2005) Synopsis of *Storckiella* Seem. (Fabaceae, Caesalpinioideae) with description of a new species and a new subspecies from New Caledonia. Adansonia 27(2): 217-230.

Nielsen IC, & Veillon J-M (2005) A new species of *Callerya* (Leguminosae, Papilionoideae, Millettieae) from New Caledonia. Adansonia 27(1): 81-84.

Pillon Y, Hopkins HCF, Bradford JC (2008) Two new species of *Cunonia* (Cunoniaceae) from New Caledonia. Kew Bulletin 63(3): 419-431. doi: 10.1007/s12225-008-9045-7.

Pillon Y, Nooteboom HP (2009) A new species of *Symplocos* (Symplocaceae) from Mont Panié (New Caledonia). Adansonia 31(1): 191-196.

Pintaud JC, Jaffré T, Puig H (2001) Chorology of New Caledonian palms and possible evidence of Pleistocene rain forest refugia. Comptes rendus de l'Académie des sciences. Série III, Sciences de la vie 324(5): 453-63. Retrieved from http://www.ncbi.nlm.nih.gov/pubmed/11411288.

Pintaud JC, Baker WJ (2008) A revision of the palm genera (Arecaceae) of New Caledonia. Kew Bulletin 63(1): 61-73. doi: 10.1007/s12225-007-9009-3.

Pintaud JC, Jaffré T, Veillon J-M (1999) Conservation status of New Caledonia palms. Pacific Conservation Biology 5: 9-15.

Pintaud JC, Jaffré T (2001) Patterns of diversity and endemism in palms on ultramafic rocks in New Caledonia. South African Journal of Science 97: 548-550.

Raynal J (1974) Notes cyperologiques. 22. Les *Costularia* de Nouvelle-Calédonie. Adansonia 14: 337-377.

Saunders RMK, Munzinger J (2007) A new species of *Goniothalamus* (Annonaceae) from New Caledonia, representing a significant range extension for the genus. Society 558: 497-503.

Schmid M (2006) Contribution à la connaissance des Myrsinaceae de Nouvelle-Calédonie. I. Le genre *Maesa* Forssk. Adansonia 28(1): 143-148.

Schmid M (2009) Contribution à la connaissance des Primulaceae (ex Myrsinaceae) de Nouvelle-calédonie. II. Le genre Rapanea Aubl. Adansonia 31(2): 341-395.

Schmid M (2012) Contribution à la connaissance des Primulaceae (ex Myrsinaceae) de Nouvelle-Calédonie. III Les genres *Tapeinosperma* Hook.F. et *Mangenotiella.* Adansonia (In press).

Snow N (2009) *Kanakomyrtus* (Myrtaceae): A new endemic genus from New Caledonia with linear stigma lobes and baccate fruits. Syst Bot 34(2): 330-344. doi: 10.1600/036364409788606253.

Swenson U, Munzinger J (2010a) Revision of *Pycnandra* subgenus *Sebertia* (Sapotaceae) and a generic key to the family in New Caledonia. Adansonia 32(2): 239-249.

Swenson U, Munzinger J (2010b) Taxonomic revision of *Pycnandra* subgenus *Trouettia* (Sapotaceae), with six new species from New Caledonia. Aust Syst Bot 23: 333-370.

Swenson U, Munzinger J (2010c) Revision of *Pycnandra* subgenus *Achradotypus* (Sapotaceae), with five new species from New Caledonia. Aust Syst Bot 23: 185-216.

Swenson U, Munzinger J, Bartish IV (2007) Molecular phylogeny of *Planchonella* (Sapotaceae) and eight new species from New Caledonia. Taxon 56(2): 329-354.

Tirel C (2009) Réhabilitation de l'espèce *Elaeocarpus coumbouiensis* Guillaumin (Elaeocarpaceae) de Nouvelle-Calédonie. Adansonia 31(1): 137-141.

Tirel C, Mcpherson G (2006) *Elaeocarpus tremulus* Tirel & McPherson, nouvelle espèce d'Elaeocarpaceae de Nouvelle-Calédonie. Adansonia 28(1): 137-141.

Venter S (2004) *Dracophyllum mackeeanum* (Ericaceae: Richeeae), a new species from New Caledonia. New Zeal J Bot 42: 747-751.

Venter S, Munzinger J (2007) *Paphia paniensis* (Ericaceae), a new species from New Caledonia critically compared with *P. neocaledonica*. New Zeal J Bot 45: 503-508.

Vink W (2003) A new species of *Zygogynum* (Winteraceae) from New Caledonia. Blumea 48: 183-186.
