# Supplementary material for: Conservation Priorities in a Biodiversity Hotspot: Analysis of Narrow Endemic Plant Species in New Caledonia
Source: PLoS One. 2013 Sep 18;8(9):e73371. doi: 10.1371/journal.pone.0073371 (PMC3776834; doi:10.1371/journal.pone.0073371)
Supplement: Appendix S2 — List of narrow endemic species (1, 2 and 3 locations). (PDF) [file pone.0073371.s002.pdf]

Appendix 2:

Narrow endemic species restricted to 1 location

Species name in bold indicates that 100% of the populations are impacted by mines. Asterisk after the species name indicates that at least 50% of records are impact by mines. N: North province; S: South province. An « X » in the different columns indicates that species are protected by local legislation/located in a protected area/impacted by mining activities.

Family	Species	IUCN status	Province occurrence	Protected by local legislation	Populations located in a protected area	Populations impacted by mining activities
Acanthaceae	*Brunoniella neocaledonica* (Heine) Moylan		N	X	-	-
**Apocynaceae**	***Alyxia veillonii* D.J.Middleton**		S	-	-	X
Apocynaceae	*Marsdenia dognyensis* Guillaumin		S	-	-	-
Apocynaceae	*Marsdenia oubatchensis* Schltr.		N	-	-	-
**Apocynaceae**	***Marsdenia variifolia* Guillaumin**		N	-	-	X
Apocynaceae	*Neisosperma sevenetii* (Boiteau) Boiteau	Endangered B1+2c ver 2.3	N	X	-	X
Apocynaceae	*Neisosperma thiollierei* (Montrouz.) Boiteau	Critically Endangered B1+2c ver 2.3	S	X	-	-
Apocynaceae	*Ochrosia bodenheimarum* Guillaumin		S	-	-	-
Apocynaceae	*Ochrosia inventorum* L. Allorge	CR A2ce	S	X	-	-
Apocynaceae	*Rauvolfia spathulata* Boiteau		S	-	X	-
Araliaceae	*Meryta rivularis* Lowry, ined.		N	-	X	-
Araliaceae	*Plerandra baillonii* (R.Vig.) Lowry, Plunkett & Frodin, ined.		N	-	-	-
Araliaceae	*Plerandra calcicola* Lowry & Plunkett, ined.		N	-	-	-
Araliaceae	*Plerandra letocartiorum* Lowry & Plunkett, ined.		S	-	-	-
Araliaceae	*Plerandra memayoensis* Lowry & Plunkett, ined.		S	-	-	-
Araliaceae	*Plerandra tronchetii* Lowry & Plunkett, ined.		N	-	-	-
Araliaceae	*Polyscias ouaiemensis* Lowry & Plunkett, ined.		N	-	-	-
Araliaceae	*Polyscias regalis* Lowry & Plunkett, ined.		N; S	-	-	-
Araliaceae	*Polyscias suprinorum* Lowry & Plunkett, ined.		ND	-	-	-
Araliaceae	*Polyscias taomensis* Lowry & Plunkett, ined.		N	X	-	-
Araucariaceae	*Agathis montana* de Laub.	Near Threatened ver 3.1	N	-	X	-
Arecaceae	*Basselinia favieri* H. E. Moore	Vulnerable D1 ver 2.3	N	X	X	-

Arecaceae	*Basselinia iterata* H. E. Moore	Vulnerable D2 ver 2.3	N	X	-	-
Arecaceae	*Basselinia vestita* H. E. Moore	Vulnerable D2 ver 2.3	N; S	X	-	-
Arecaceae	*Burretiokentia dumasii* Pintaud & Hodel		S	X	X	-
Arecaceae	*Burretiokentia grandiflora* Pintaud & Hodel		S	X	X	-
Arecaceae	*Burretiokentia koghiensis* Pintaud & Hodel		S	X	-	-
Arecaceae	*Clinosperma macrocarpa* (H. E. Moore) Pintaud & W. J.Baker	Critically Endangered D ver 2.3	N	X	X	-
Arecaceae	*Cyphophoenix elegans* (Brongn. & Gris) H. Wendl. ex Salomon	Vulnerable B1+2c ver 2.3	N	X	-	-
Arecaceae	*Cyphophoenix nucele* H. E. Moore	Critically Endangered B1+2c ver 2.3	L	X	-	-
Arecaceae	*Kentiopsis magnifica* (H. E. Moore) Pintaud & Hodel	Vulnerable B1+2c ver 2.3	N	X	-	-
Arecaceae	*Pritchardiopsis jeanneneyi* Becc.	Critically Endangered D ver 2.3	S	X	X	-
Brassicaceae	*Rorippa neocaledonica* Jonsell		S	-	-	-
Burseraceae	*Canarium trifoliolatum* Engl.		N	-	-	-
Celastraceae	*Elaeodendron parvifolium* R. H. Archer		N	X	-	X
Chrysobalanaceae	*Hunga cordata* Prance	Endangered B1+2c ver 2.3	N	X	-	X
Cunoniaceae	*Codia triverticillata* H. C. Hopkins & Pillon		N	X	-	X
Cunoniaceae	*Cunonia x koghicola* H. C. Hopkins, J. Bradford & Pillon		S	-	-	X
Cunoniaceae	*Geissois belema* Pillon & H.C. Hopkins, ined.		N	-	-	-
Cunoniaceae	*Geissois bradfordii* H.C.Hopkins		S	X	X	-
Cunoniaceae	*Hooglandia ignambiensis* McPherson & Lowry		N	X	-	-
Cunoniaceae	*Pancheria minima* J.C. Bradford		N	X	X	-
Cunoniaceae	*Pancheria ouaiemensis* J.C. Bradford	Vulnerable D1 ver 2.3	N	X	-	-
Cunoniaceae	*Pancheria x heterophylla* Veill. ex Guillaumin		S	-	-	-
Cunoniaceae	*Pancheria x lanceolata* (Pamp.) Baker f.		N	-	-	-
Cunoniaceae	*Weinmannia ouaiemensis* (Guillaumin & Virot) Hoogland		N	X	-	-
Cyperaceae	*Baumea veillonis* J. Raynal		S	-	X	X
Dilleniaceae	*Hibbertia bouletii* Veillon		S	-	-	-
Dilleniaceae	*Hibbertia favieri* Veillon *		S	-	-	X
Dilleniaceae	*Hibbertia margaretae* Veillon		S	-	-	-
Dilleniaceae	*Hibbertia rubescens* Vieill. ex Guillaumin		N	X	-	X

Ebenaceae	*Diospyros fastidiosa* F. White	Vulnerable D2 ver 2.3	N	-	X	-
Ebenaceae	*Diospyros inexplorata* F. White		S	-	-	-
Ebenaceae	*Diospyros nebulosa* F. White	Vulnerable D2 ver 2.3	N	-	X	-
Ebenaceae	*Diospyros neglecta* F. White		N	-	-	-
Ebenaceae	*Diospyros tridentata* F. White		N	-	-	-
Ebenaceae	*Diospyros trisulca* F. White	Vulnerable D1 ver 2.3	N	-	-	-
Ebenaceae	*Diospyros veillonii* F. White	Critically Endangered D ver 2.3	S	X	-	-
Elaeocarpaceae	*Elaeocarpus biflorus* Tirel		N	-	X	-
Elaeocarpaceae	*Elaeocarpus castanaefolius* Guillaumin		S	-	-	-
Elaeocarpaceae	*Elaeocarpus tremulus* Tirel & McPherson *		S	-	X	X
**Elaeocarpaceae**	***Sloanea billardieri* (Vieill.) A. C. Smith**		N	-	-	X
Elaeocarpaceae	*Sloanea lepida* Tirel	Vulnerable D1 ver 2.3	S	-	X	-
Elaeocarpaceae	*Sloanea suaveolens* Tirel	Vulnerable D1 ver 2.3	N	-	-	-
Ericaceae	*Dracophyllum ouaiemense* Virot		N	-	-	-
Ericaceae	*Paphia neocaledonica* (Guillaumin) P.F. Stevens		N; S	X	-	-
Ericaceae	*Paphia paniensis* S.Venter & Munzinger		N	X	X	-
Euphorbiaceae	*Alphandia resinosa* Baill.		N	X	-	-
Euphorbiaceae	*Baloghia pininsularis* Guillaumin	Endangered B1+2c ver 2.3	S	X	-	-
**Euphorbiaceae**	***Baloghia pulchella* Schltr.**		S	-	-	X
Euphorbiaceae	*Bocquillonia arborea* Airy Shaw	Endangered B1+2c ver 2.3	S	X	-	-
Euphorbiaceae	*Bocquillonia codonostylis* (Baill.) Airy Shaw		N	-	X	-
Euphorbiaceae	*Bocquillonia phenacostigma* Airy Shaw		N	-	X	-
Euphorbiaceae	*Trigonostemon cherrieri* Veillon	CR B1ab(iii)+2ab(iii)	S	X	-	-
Fabaceae	*Callerya neocaledonica* I.C. Nielsen & Veillon		S	-	-	-
Fabaceae	*Canavalia favieri* I.C.Nielsen		S	-	-	-
Fabaceae	*Canavalia veillonii* I.C.Nielsen	CR A2ce	S	-	-	-
Fabaceae	*Serianthes germainii* Guillaumin		S	X	-	-
Fabaceae	*Serianthes lifouensi*s (Fosberg) I.C.Nielsen		S	-	-	-
**Goodeniaceae**	***Scaevola barrierei* Wulff & Munzinger ined.**		N	-	-	X
Goodeniaceae	*Scaevola coccinea* Däniker *		S	-	-	X

Goodeniaceae	*Scaevola racemigera* Daeniker		S	-	X	-
Iridaceae	*Patersonia neocaledonia* Goldblatt & J.C.Manning, ined		S	-	X	-
Lamiaceae	*Gmelina lignum-vitreum* Guillaumin	Critically Endangered D ver 2.3	S	X	-	-
Lauraceae	*Adenodaphne macrophylla* Kosterm.		N	-	-	-
**Lauraceae**	***Cryptocarya bitriplinervia* Kosterm.**	Endangered B1+2c ver 2.3	S	X	-	X
Lauraceae	*Litsea imbricata* Guillaumin	Endangered B1+2c ver 2.3	N	X	-	-
Lauraceae	*Litsea mackeei* Kosterm.		N	X	-	X
Lauraceae	*Litsea ovalis* Kosterm.		N	-	-	-
Lauraceae	*Litsea paouensis* Guillaumin		N	-	-	-
**Lauraceae**	***Litsea racemiflora* Däniker**		N	-	-	X
Lauraceae	*Litsea stenophylla* Guillaumin		S	-	-	-
Malpighiaceae	*Stigmaphyllon mcphersonii* C.E. Anderson *		S	-	-	X
Malvaceae	*Acropogon aoupiniensis* Morat	Vulnerable D2 ver 2.3	N	-	X	-
Malvaceae	*Acropogon calcicolus* Morat & Chalopin	EN B1ab(iii)+2ab(iii)	N	X	-	-
Malvaceae	*Acropogon chalopiniae* Morat		S	-	X	-
Malvaceae	*Acropogon fatsioides* Schltr.	Vulnerable B1+2c ver 2.3	N	-	-	-
Malvaceae	*Acropogon megaphyllus* (Bureau & J. Poiss. ex Guillaumin) Morat	Vulnerable D2 ver 2.3	S	-	-	-
Malvaceae	*Acropogon paagoumensis* Morat & Chalopin		N	X	-	X
Malvaceae	*Acropogon tireliae* Morat & Chalopin		N	-	-	-
Myodocarpaceae	*Myodocarpus touretteorum* Lowry, ined.		S	-	-	X
Myrtaceae	*Eugenia belepiana* J.W.Dawson, ined.		N	-	-	-
Myrtaceae	*Eugenia daenikeri* Guillaumin	Endangered B1+2c ver 2.3	S	X	X	-
Myrtaceae	*Eugenia excorticata* J.W.Dawson, ined.		S	-	-	-
Myrtaceae	*Eugenia gomonenensis* (Guillaumin) J.W.Dawson, comb. ined.		N	-	-	-
Myrtaceae	*Eugenia grisiana* Guillaumin		N	-	-	-
Myrtaceae	*Eugenia munzingeri* J.W.Dawson, ined.		S	-	-	-
Myrtaceae	*Eugenia nekoroensis* J.W.Dawson, ined.		N	-	-	-
Myrtaceae	*Eugenia neo-caledonica* (Brongn. & Gris) J. W. Dawson		N	-	-	-
Myrtaceae	*Eugenia poindimiensis* J.W.Dawson, ined.		N	-	-	-

Myrtaceae	*Eugenia styphelioides* (Schltr.) J.W.Dawson, comb. ined. *		S	-	-	X
**Myrtaceae**	***Eugenia taomensis* J.W.Dawson, ined.**		N	-	-	X
Myrtaceae	*Eugenia tiwakensis* J.W.Dawson, ined.		N	-	-	-
Myrtaceae	*Eugenia veluticarpa* J.W.Dawson, ined.		N	-	-	-
**Myrtaceae**	***Gossia katepahiensis* N. Snow, ined.**		N	-	-	X
Myrtaceae	*Gossia mandjeliaensis* N. Snow, ined.		N	-	-	-
Myrtaceae	*Gossia ngaensis* N. Snow, ined.		S	-	-	-
**Myrtaceae**	***Gossia ouazangouensis* N. Snow, ined.**		N	-	-	X
Myrtaceae	*Kanakomyrtus dawsoniana* N.Snow		N	-	-	-
Myrtaceae	*Metrosideros cherrieri* J. W. Dawson		N	-	-	-
Myrtaceae	*Metrosideros longipetiolata* J.W.Dawson		N	-	-	-
Myrtaceae	*Metrosideros rotundifolia* J.W.Dawson		N	-	-	-
Myrtaceae	*Metrosideros whitakeri* J.W.Dawson		N	-	-	-
Myrtaceae	*Syzygium aoupinianum* J. W. Dawson		N	-	X	-
Myrtaceae	*Syzygium brevipes* (Brongniart & Gris) J. W. Dawson		N	-	-	-
Myrtaceae	*Syzygium filiflorum* J. W. Dawson		N	-	-	-
Myrtaceae	*Syzygium koumacense* J. W. Dawson		N	-	-	-
**Myrtaceae**	***Syzygium laxeracemosum* (Guillaumin) J. W. Dawson**		S	-	-	X
Myrtaceae	*Syzygium nanum* J. W. Dawson		S	-	-	-
Myrtaceae	*Syzygium parvicarpum* J. W. Dawson		S	-	-	-
Myrtaceae	*Syzygium sarmentosum* J. W. Dawson		N	-	-	-
Myrtaceae	*Syzygium tchambaense* J. W. Dawson		N	-	-	-
**Myrtaceae**	***Syzygium virotii* J. W. Dawson**		S	-	-	X
Myrtaceae	*Tristaniopsis jaffrei* J.W. Dawson		N	X	-	X
Myrtaceae	*Tristaniopsis lucida* J.W. Dawson	Lower Risk/conservation dependent ver 2.3	S	-	X	-
Myrtaceae	*Tristaniopsis minutiflora* J.W. Dawson	Vulnerable B1+2c ver 2.3	N	X	-	-
Myrtaceae	*Tristaniopsis ninndoensis* J.W. Dawson		N	-	-	-
Myrtaceae	*Tristaniopsis yateensis* J. W. Dawson	Endangered B1+2c ver 2.3	S	X	X	-
**Myrtaceae**	***Xanthostemon francii* Guillaumin**		S	-	-	X

Myrtaceae	*Xanthostemon lateriflorus* Guillaumin		N	-	-	-
**Myrtaceae**	***Xanthostemon longipes* Guillaumin**		S	-	-	X
Oleaceae	*Jasminum promunturianum* Däniker		N	X	-	-
Orchidaceae	*Acianthus bracteatus* Rendle		S	X	-	-
Orchidaceae	*Acianthus corniculatus* Rendle		N	X	-	-
Orchidaceae	*Acianthus macroglossus* Schltr.		S	X	-	-
Orchidaceae	*Acianthus uvarius* N. Hallé		S	X	-	X
Orchidaceae	*Acianthus veillonis* N. Hallé		S	X	-	-
Orchidaceae	*Bulbophyllum lophoglottis* (Guillaumin) N. Hallé		S	X	-	X
Orchidaceae	*Habenaria insularis* Schltr.		N	X	-	-
Orchidaceae	*Megastylis paradoxa* (Kraenzl.) N. Hallé		S	X	X	-
Orchidaceae	*Octarrhena saccolabioides* (Schltr.) Schltr.		N	X	-	-
Orchidaceae	*Peristylus minimiflorus* (Kraenzl.) N. Hallé		S	X	-	-
Oxalidaceae	*Oxalis elsae* Knuth		N	-	-	-
Pandanaceae	*Pandanus cavatus* H. St. John		N	-	-	-
**Pandanaceae**	***Pandanus decastigma* Stone**	Vulnerable B1+2c ver 2.3	S	-	-	X
Pandanaceae	*Pandanus globatus* H. St. John		N	-	-	-
Phyllanthaceae	*Phyllanthus amieuensis* Guillaumin		N	-	-	-
Phyllanthaceae	*Phyllanthus aoupinieensis* M. Schmid		N	-	X	-
Phyllanthaceae	*Phyllanthus artensis* M. Schmid		N	-	-	-
Phyllanthaceae	*Phyllanthus avanguiensis* M. Schmid		N	X	-	X
Phyllanthaceae	*Phyllanthus casearoides* S. Moore		N	-	-	-
Phyllanthaceae	*Phyllanthus cherrieri* M. Schmid		N	-	-	-
Phyllanthaceae	*Phyllanthus comptonii* S. Moore		S	-	-	-
Phyllanthaceae	*Phyllanthus cornutus* Baill.		S	-	-	-
Phyllanthaceae	*Phyllanthus deciduiramus* Däniker		N	X	-	X
Phyllanthaceae	*Phyllanthus dumbeaensis* M. Schmid		S	-	-	-
Phyllanthaceae	*Phyllanthus fractiflexus* M. Schmid		N	X	-	X
Phyllanthaceae	*Phyllanthus golonensis* M. Schmid		N	-	-	-
Phyllanthaceae	*Phyllanthus guillauminii* Däniker		N	X	-	X

Phyllanthaceae	*Phyllanthus houailouensis* M. Schmid		N	-	-	-
Phyllanthaceae	*Phyllanthus koghiensis* Guillaumin		S	-	X	-
Phyllanthaceae	*Phyllanthus macrochorion* Baillon		N	-	-	-
Phyllanthaceae	*Phyllanthus mandjeliaensis* M. Schmid		N	-	-	-
Phyllanthaceae	*Phyllanthus mangenotii* M. Schmid		N	-	-	X
Phyllanthaceae	*Phyllanthus margaretae* M. Schmid		N	-	X	-
Phyllanthaceae	*Phyllanthus mcphersonii* M. Schmid		N	-	-	-
Phyllanthaceae	*Phyllanthus meuieensis* M. Schmid		N	-	-	-
Phyllanthaceae	*Phyllanthus natoensis* M. Schmid		N	-	-	-
Phyllanthaceae	*Phyllanthus nitens* M. Schmid		N	X	-	-
Phyllanthaceae	*Phyllanthus nothisii* M. Schmid		N	X	-	X
**Phyllanthaceae**	***Phyllanthus parangoyensis* M. Schmid**		N	-	-	X
Phyllanthaceae	*Phyllanthus paucitepalus* M. Schmid		S	-	-	-
Phyllanthaceae	*Phyllanthus petchikaraensis* M. Schmid *		S	-	-	X
Phyllanthaceae	*Phyllanthus pindaiensis* M. Schmid	CR B1ab(iii)+2ab(iii)	N	X	-	-
Phyllanthaceae	*Phyllanthus pinjenensis* M. Schmid		N	-	-	-
Phyllanthaceae	*Phyllanthus rhodocladus* S. Moore		N	-	-	-
Phyllanthaceae	*Phyllanthus rozennae* M. Schmid		N	-	-	-
Phyllanthaceae	*Phyllanthus salicifolius* Baill.		N	-	-	-
Phyllanthaceae	*Phyllanthus stenophyllus* Guillaumin		N	-	-	-
Phyllanthaceae	*Phyllanthus stipitatus* M. Schmid		N	X	-	-
Phyllanthaceae	*Phyllanthus tangoensis* M. Schmid		N	-	-	-
Phyllanthaceae	*Phyllanthus tiebaghiensis* M. Schmid		N	X	-	X
Phyllanthaceae	*Phyllanthus tixieri* M. Schmid		N	-	-	X
Phyllanthaceae	*Phyllanthus trichopodus* Guillaumin		N	-	-	-
**Phyllanthaceae**	***Phyllanthus tritepalus* M. Schmid**		N	-	-	X
Phyllanthaceae	*Phyllanthus unioensis* M. Schmid		S	-	-	-
Phyllanthaceae	*Phyllanthus veillonii* M. Schmid		N	-	-	-
Phyllanthaceae	*Phyllanthus virgultiramus* Däniker		N	X	-	X
Picrodendraceae	*Austrobuxus cracens* McPherson	Vulnerable D1 ver 2.3	N	-	-	-

Picrodendraceae	*Austrobuxus mandjelicus* McPherson		N	-	-	-
**Picrodendraceae**	***Austrobuxus montis-do* Airy Shaw**	Lower Risk/conservation dependent ver 2.3	S	-	X	X
Pittosporaceae	*Pittosporum bernardii* Tirel & Veillon		N	-	-	-
Pittosporaceae	*Pittosporum lanipetalum* Tirel & Veillon		N	-	-	-
Pittosporaceae	*Pittosporum leroyanum* Tirel & Veillon		S	-	-	-
Pittosporaceae	*Pittosporum ornatum* Tirel & Veillon	Endangered B1+2c ver 2.3	S	X	-	-
Pittosporaceae	*Pittosporum paniense* Guillaumin	Vulnerable D2 ver 2.3	N	X	X	-
Pittosporaceae	*Pittosporum sylvaticum* Guillaumin		S	-	X	X
Pittosporaceae	*Pittosporum tanianum* Veillon & Tirel	Critically Endangered D ver 3.1	S	X	X	-
Poaceae	*Lepturopetium kuniense* Morat		S	-	-	-
Podocarpaceae	*Dacrydium guillauminii* J.Buchholz		S	X	-	X
Podocarpaceae	*Podocarpus colliculatus* (N.E. Gray) de Laub.	Critically Endangered B1ab(iii,v);C2a(i) ver 3.1	S	-	-	-
**Primulaceae**	***Rapanea albiflorens* M. Schmid**		N	-	-	X
Primulaceae	*Rapanea boulindaensis* M. Schmid		N	-	-	X
Primulaceae	*Rapanea dumbeaensis* M.Schmid		S	-	-	-
Primulaceae	*Rapanea munzingeri* M. Schmid		S	-	X	-
Primulaceae	*Rapanea ouazangouensis* M. Schmid		N	-	-	-
Primulaceae	*Rapanea paniensis* M. Schmid		N	-	X	-
**Primulaceae**	***Rapanea poumensis* M. Schmid**		N	-	-	X
Primulaceae	*Rapanea pronyensis* Guillaumin		S	-	-	-
Primulaceae	*Rapanea spissifolia* M. Schmid		N	-	-	X
Primulaceae	*Rapanea tchingouensis* M. Schmid		N	-	-	-
Primulaceae	*Rapanea yateensis* M. Schmid		S	-	X	-
Primulaceae	*Tapeinosperma amossense* Guillaumin		N	-	-	-
Primulaceae	*Tapeinosperma colnettianum* Guillaumin		N	-	X	-
Primulaceae	*Tapeinosperma golonense* M. Schmid, ined.		N	-	-	-
Primulaceae	*Tapeinosperma kaalensis* M.Schmid, ined.		N	-	-	-
Primulaceae	*Tapeinosperma paniense* M.Schmid, ined.		N	-	X	-
Primulaceae	*Tapeinosperma pauciflorum* Mez		N	-	-	-
Primulaceae	*Tapeinosperma pulchellum* Mez		N	-	-	-

Primulaceae	*Tapeinosperma storezii* M.Schmid, ined.		N	-	X	-
Primulaceae	*Tapeinosperma veillonii* M.Schmid, ined.		N	-	-	-
Primulaceae	*Tapeinosperma whitei* Guillaumin		S	-	-	-
Proteaceae	*Beauprea penariensis* Guillaumin		S	-	-	-
Proteaceae	*Virotia angustifolia* (Virot) P. H. Weston & A. R. Mast		N	X	-	X
Rubiaceae	*Guettarda artensis* Guillaumin		N	-	-	-
Rubiaceae	*Ixora lecardii* Guillaumin		S	-	-	-
Rubiaceae	*Morinda truncata* J.T. Johanss.		N	X	-	X
Rubiaceae	*Psychotria amieuensis* Guillaumin		S	-	X	-
Rubiaceae	*Psychotria calliantha* (Baill.) Guillaumin		S	-	-	-
Rubiaceae	*Psychotria pininsularis* Guillaumin		S	-	-	-
Rubiaceae	*Psychotria speciosa* (Montrouz.) S. Moore		N	-	-	-
Rubiaceae	*Psychotria unioensis* Guillaumin		S	-	-	-
Rubiaceae	*Randia baladica* Montrouz. Ex Guillaumin		N	-	-	-
Rubiaceae	*Thiollierea dagostinii* Barrabe & Mouly, ined.		N	X	-	-
Rubiaceae	*Thiollierea kaalaensis* (N.Hallé & Jérémie) Barrabé & Mouly, comb. ined.		N	X	-	X
Rubiaceae	*Thiollierea rigaultii* Barrabe & Mouly, ined.		N	X	-	-
Rubiaceae	*Tinadendron noumeanum* (Baill.) Achille	CR A2ce	S	X	-	-
Rutaceae	*Boronella koniamboensis* (Däniker) T.G.Hartley	Vulnerable B1+2c ver 2.3	N	X	-	X
Rutaceae	*Comptonella glabra* T.G. Hartley		N	X	-	X
Rutaceae	*Crossosperma cauliflora* T.G. Hartley		N	X	X	-
Rutaceae	*Medicosma articulata* T.G.Hartley		N	-	-	-
**Rutaceae**	***Medicosma congesta* T.G.Hartley**		N	-	-	X
**Rutaceae**	***Medicosma diversifolia* T.G.Hartley**		N	-	-	X
Rutaceae	*Medicosma latifolia* T.G.Hartley		N	-	-	-
Rutaceae	*Medicosma obliqua* T.G.Hartley		S	-	-	-
Rutaceae	*Medicosma parvifolia* T.G.Hartley *		S	-	-	X
Rutaceae	*Medicosma petiolaris* T.G.Hartley		N	-	-	-
Rutaceae	*Medicosma suberosa* T.G.Hartley		S	-	-	-
**Rutaceae**	***Medicosma tahafeana* T.G.Hartley**		N	-	-	X

Rutaceae	*Oxanthera aurantium* Tanaka *	Vulnerable B1+2c ver 2.3	N	-	-	X
Rutaceae	*Oxanthera fragrans* Montrouz.	Endangered B1+2c ver 2.3	N	X	-	-
Rutaceae	*Oxanthera undulata* (Guillaumin) Swingle	Critically Endangered D ver 2.3	S	X	-	-
Rutaceae	*Sarcomelicope glauca* T.G.Hartley	Critically Endangered B1+2c ver 2.3	S	X	-	-
Rutaceae	*Sarcomelicope megistophylla* T.G.Hartley		N	-	-	-
Rutaceae	*Zanthoxylum leratii* Guillaumin		S	-	X	-
Rutaceae	*Zieria chevalieri* Virot	Vulnerable B1+2c ver 2.3	N	X	-	X
Salicaceae	*Homalium betulifolium* Däniker	Endangered B1+2c ver 2.3	S	X	-	X
Salicaceae	*Homalium buxifolium* Däniker	Endangered B1+2c ver 2.3	N	X	-	-
Salicaceae	*Homalium rubrocostatum* Sleumer	Endangered B1+2c ver 2.3	N	X	-	X
Salicaceae	*Homalium sleumerianum* Lescot	Vulnerable B1+2c ver 2.3	N	-	-	-
Salicaceae	*Lasiochlamys hurlimannii* (Guillaumin) Sleumer	Endangered B1+2c ver 2.3	S	X	-	-
Salicaceae	*Lasiochlamys pseudocoriacea* Sleumer	Vulnerable D2 ver 2.3	N	-	-	-
Salicaceae	*Xylosma gigantifolium* Sleumer		ND	-	-	-
Salicaceae	*Xylosma inaequinervium* Sleumer	Endangered B1+2c ver 2.3	N	X	-	X
Salicaceae	*Xylosma peltatum* (Sleumer) Lescot	Critically Endangered B1+2c ver 2.3	S	X	-	-
Sapindaceae	*Cupaniopsis crassivalvis* Radlk.	Extinct ver 2.3	S	-	-	-
Sapindaceae	*Cupaniopsis grisea* Adema		N	-	-	-
Sapindaceae	*Cupaniopsis mouana* Guillaumin	Endangered B1+2c ver 2.3	S	X	-	-
Sapindaceae	*Cupaniopsis rosea* Adema	Endangered B1+2c ver 2.3	N	X	-	-
Sapindaceae	*Cupaniopsis rotundifolia* Adema	Endangered B1+2c ver 2.3	S	X	-	-
Sapindaceae	*Cupaniopsis tontoutensis* Guillaumin	Endangered B1+2c ver 2.3	S	X	-	X
**Sapindaceae**	***Elattostachys dzumacensis* Adema**	Vulnerable D1 ver 2.3	S	-	-	X
Sapotaceae	*Planchonella crenata* Munzinger & Swenson		N	X	-	X
**Sapotaceae**	***Planchonella ericiflora* Munzinger & Swenson**		S	-	-	X
Sapotaceae	*Planchonella latihila* Munzinger & Swenson		S	X	-	X
**Sapotaceae**	***Planchonella rheophytopsis* P. Royen**		N	-	-	X
Sapotaceae	*Planchonella skottsbergii* Gauillaumin		S	-	-	-
Sapotaceae	*Pycnandra belepensis* Swenson & Munzinger		N	-	-	-
Sapotaceae	*Pycnandra blaffartii* Swenson & Munzinger		N	-	X	-

Sapotaceae	*Pycnandra bourailensis* Swenson & Munzinger		S	-	-	-
Sapotaceae	*Pycnandra confusa* Swenson & Munzinger		S	-	-	-
Sapotaceae	*Pycnandra goroensis* (Aubrév.) Munzinger & Swenson, comb. ined.	Critically Endangered D ver 2.3	S	X	-	-
Sapotaceae	*Pycnandra linearifolia* Swenson & Munzinger		N	-	-	-
Sapotaceae	*Pycnandra longiflora* (Benth.) Munzinger & Swenson, comb. ined.		N	-	-	-
Sapotaceae	*Pycnandra longipetiolata* Swenson & Munzinger		N	-	-	-
Sapotaceae	*Pycnandra multiflora* (Vink) Munzinger & Swenson , comb. ined.	Vulnerable B1+2c ver 2.3	N	-	-	-
Sapotaceae	*Pycnandra ouaiemensis* Swenson & Munzinger		N	-	-	-
Simaroubaceae	*Soulamea cycloptera* Guillaumin		N	-	-	-
Simaroubaceae	*Soulamea dagostinii* Jaffré & Fambart		N	X	-	-
**Simaroubaceae**	***Soulamea moratii* Jaffré & Fambart**		N	-	-	X
Simaroubaceae	*Soulamea rigaultii* Jaffré & Fambart		N	X	-	X
Solanaceae	*Solanum insulae-pinorum* Heine		S	-	-	-
Solanaceae	*Solanum pseuderanthemoides* Schltr.		S	-	-	-
Solanaceae	*Solanum vaccinioides* Schltr.		N	X	-	X
Symplocaceae	*Symplocos paniensis* Pillon & Noot.		N	-	X	-
Violaceae	*Agatea lecointei* Munzinger		N	-	-	-
Winteraceae	*Zygogynum oligostigma* Vink	Endangered B1+2c ver 2.3	N	X	-	X
Winteraceae	*Zygogynum pauciflorum* (Baker f.) Vink		N	-	X	-
Winteraceae	*Zygogynum tanyostigma* Vink	Vulnerable B1+2c ver 2.3	N	-	X	-

Narrow endemic species restricted to 2 locations

Species name in bold indicates that 100% of the populations are impacted by mines. Asterisks after the species name indicates that at least 50% of records are impact by mines. N: North province; S: South province. An « X » in the different columns indicates that species are protected by local legislation/located in a protected area/impacted by mining activities.

Family	Species	IUCN status	Province occurrence	Protected by local legislation	Populations located in a protected area	Populations impacted by mining activities
Acanthaceae	*Graptophyllum ophiolithicum* Heine		N	X	-	X
Anacardiaceae	*Semecarpus poyaensis* M.Hoff		N	X	-	-
Apocynaceae	*Marsdenia speciosa* Baill.		S	-	X	-
Apocynaceae	*Marsdenia tylophoroides* Schltr.		N; S	-	X	-
Apocynaceae	*Parsonsia terminaliifolia* Guillaumin *		N	-	-	X
Apocynaceae	*Rauvolfia sevenetii* Boiteau		S	X	X	X
Araliaceae	*Plerandra longistyla* Lowry, Plunkett & Frodin *		N	-	-	X
Araliaceae	*Polyscias mackeei* Lowry & Plunkett		S	-	X	-
Araliaceae	*Polyscias otopyrena* (Baill.) Lowry & Plunkett		S	-	-	-
Araucariaceae	*Araucaria nemorosa* de Laub.	Critically Endangered B1ab(i,ii,iii,iv,v)+2ab(i,ii,iii,iv,v) ver 3.1	S	X	-	-
Araucariaceae	*Araucaria schmidii* de Laub.	Vulnerable D2 ver 3.1	N	-	X	-
Arecaceae	*Basselinia porphyrea* H. E. Moore	Lower Risk/conservation dependent ver 2.3	S	X	X	-
Arecaceae	*Basselinia tomentosa* Becc.	Vulnerable D2 ver 2.3	S	X	-	-
Arecaceae	*Burretiokentia hapala* H. E. Moore		N	X	-	-
Arecaceae	*Kentiopsis piersoniorum* Pintaud & Hodel		N	-	X	-
Balanopaceae	*Balanops oliviformis* Baill.		N; S	-	X	-
Casuarinaceae	*Casuarina teres* Schltr.		N	X	-	X
Celastraceae	*Menepetalum salicifolium* Loes.		N	-	-	-
Celastraceae	*Salaciopsis longistyla* I. H. Müller		N	-	-	-
Chrysobalanaceae	*Hunga myrsinoides* (Schltr.) Prance		N	-	-	-
Combretaceae	*Terminalia cherrieri* MacKee	EN A2ce+3e	N; S	X	-	-
Cunoniaceae	*Codia fusca* (Schltr.) H.C. Hopkins		S	-	X	-
Cunoniaceae	*Codia microphylla* Vieill. ex Guillaumin		N	X	-	-
Cunoniaceae	*Cunonia dickisonii* Pillon & H. C. Hopkins		S	-	X	-
Cunoniaceae	*Cunonia pseudoverticillata* Guillaumin		S	-	X	-

Cunoniaceae	*Cunonia rotundifolia* Däniker	Lower Risk/conservation dependent ver 2.3	S	X	X	-
Cunoniaceae	*Cunonia rupicola* Hoogland		N	-	X	-
Cunoniaceae	*Pancheria humboldtiana* Guillaumin ex H.C.Hopkins & J.Bradford	Lower Risk/conservation dependent ver 2.3	S	-	X	-
Cunoniaceae	*Pancheria robusta* Guillaumin	Lower Risk/conservation dependent ver 2.3	S	-	X	-
Cunoniaceae	*Pancheria rubrivenia* Baker f.		N	-	-	-
Cupressaceae	*Libocedrus chevalieri* J. Buchholz	Critically Endangered B1ab(iii) ver 3.1	S	X	X	-
Cyperaceae	*Schoenus microcephalus* J. Kern		N	-	-	-
Dilleniaceae	*Hibbertia moratii* Veillon		N	X	-	X
Ebenaceae	*Diospyros erudita* F. White		N	X	-	X
Elaeocarpaceae	*Dubouzetia guillauminii* Virot		S	-	X	-
Elaeocarpaceae	*Elaeocarpus colnettianus* Guillaumin	Vulnerable D1 ver 2.3c	N	-	-	-
Ericaceae	*Dracophyllum alticola* Däniker		N	-	X	-
Ericaceae	*Styphelia enervia* (Guillaumin) Sleumer *		S	-	-	X
Escalloniaceae	*Polyosma discolor* Baill.		N	-	-	-
Euphorbiaceae	*Baloghia balansae* (Baill.) Pax		N	-	-	-
Euphorbiaceae	*Bocquillonia castaneifolia* Guillaumin		N; S	X	-	X
Euphorbiaceae	*Cleidion lemurum* McPherson	Endangered B1+2c ver 2.3	N	X	-	-
Euphorbiaceae	*Cleidion veillonii* McPherson	Critically Endangered D ver 2.3	N	X	-	-
Euphorbiaceae	*Croton cordatulus* Airy Shaw	Vulnerable D2 ver 2.3	N	X	-	X
Fabaceae	*Serianthes margaretae* I.C.Nielsen	Endangered B1+2c ver 2.3	N	-	-	X
Goodeniaceae	*Scaevola macropyrena* I.H. Muller	Vulnerable C2a ver 2.3	N	X	-	-
Lamiaceae	*Gmelina tholicola* Mabb.		S	X	X	-
Lamiaceae	*Oxera microcalyx* Guillaumin		N	-	-	X
Lamiaceae	*Oxera rugosa* Guillaumin	Vulnerable B1+2c ver 2.3	S	X	-	-
**Lauraceae**	***Adenodaphne triplinervia* Kosterm.**		S	-	X	-
Lauraceae	*Cryptocarya schmidii* Kosterm.		N	-	-	X
Lauraceae	*Litsea humboldtiana* Guillaumin		L; S	X	-	-
Lauraceae	*Litsea pentaflora* Guillaumin		N	X	-	X
Loganiaceae	*Logania imbricata* (Guillaumin) Steenis & Leenh.		N	X	-	-
Malvaceae	*Acropogon bosseri* Morat & Chalopin *		S	-	X	-

Malvaceae	*Acropogon jaffrei* Morat & Chalopin		S	X	X	X
Malvaceae	*Acropogon macrocarpus* Morat & Chalopin		N	-	-	X
Malvaceae	*Acropogon margaretae* Morat & Chalopin		N	-	X	-
Menispermaceae	*Hypserpa mackeei* Forman		N	X	-	-
Monimiaceae	*Hedycarya aragoensis* Jérémie		S	X	-	X
Myrtaceae	*Archirhodomyrtus vieillardii* (Brongn. & Gris) Burret *		N	-	-	-
Myrtaceae	*Eugenia gatopensis* Gauillaumin		S	X	-	X
**Myrtaceae**	***Eugenia jaffrei* J.W.Dawson, ined.**	Vulnerable B1+2c ver 2.3	N	-	-	-
Myrtaceae	*Eugenia lepredourii* J.W.Dawson, ined.		N	X	-	X
Myrtaceae	*Eugenia metzdorfii* J.W.Dawson, ined.	CR A2ce	S	X	X	-
Myrtaceae	*Eugenia ouaiemensis* J.W.Dawson, ined.	EN B1ab(iii)+2ab(iii)	N; S	-	-	-
Myrtaceae	*Eugenia poimbailensis* (Guillaumin) J.W.Dawson, comb. ined.		N	-	X	-
Myrtaceae	*Eugenia sicafolia* J.W.Dawson, ined.		N	-	-	-
Myrtaceae	*Eugenia tchambensis* J.W.Dawson, ined.		N	-	-	-
Myrtaceae	*Gossia colnettiana* (Guillaumin) N. Snow, comb. Ined.		N	-	-	-
Myrtaceae	*Gossia grandiflora* N. Snow, ined.		N	-	-	-
Myrtaceae	*Gossia kuakuense* (Vieill. ex Guillaumin) N. Snow, comb. ined.		N	-	X	-
Myrtaceae	*Kanakomyrtus mcphersonii* N.Snow		N; S	-	-	-
Myrtaceae	*Kanakomyrtus prominens* N.Snow		N	-	X	-
Myrtaceae	*Kanakomyrtus revoluta* N.Snow		N	-	X	-
Myrtaceae	*Melaleuca brevisepala* (J. W. Dawson) Craven & J.W. Dawson		N	-	-	-
Myrtaceae	*Metrosideros patens* J.W.Dawson		N	-	-	-
Myrtaceae	*Metrosideros tetrasticha* Guillaumin		S	-	X	-
Myrtaceae	*Syzygium apetiolatum* J. W. Dawson		S	-	X	-
Myrtaceae	*Syzygium brevioperculatum* J. W. Dawson		N	-	X	-
Myrtaceae	*Syzygium micans* Brongn. & Gris		N	-	X	-
Myrtaceae	*Syzygium nitens* J. W. Dawson		N	-	X	-
Myrtaceae	*Syzygium pendulinum* J. W. Dawson		S	X	X	X
Myrtaceae	*Syzygium ramilepis* J. W. Dawson	EN B1ab(iii)+2ab(iii)	S	-	-	-
**Myrtaceae**	***Syzygium tontoutaense* J. W. Dawson**		N	-	X	-

Myrtaceae	*Syzygium veillonii* J. W. Dawson		S	-	-	X
Myrtaceae	*Tristaniopsis polyandra* (Guillaumin) PeterG.Wilson & J.T.Waterh.	EN B1ab(iii)+2ab(iii)	S	X	-	-
Myrtaceae	*Uromyrtus nekouana* (Guillaumin) Burret	Endangered B1+2c ver 2.3	S	X	-	X
Myrtaceae	*Uromyrtus sunshinensis* (Guillaumin) N. Snow & Guymer		N	-	-	-
Myrtaceae	*Uromyrtus thymifolia* (Guillaumin) Burret		S	-	-	-
Myrtaceae	*Xanthostemon glaucus* Pamp.		S	X	-	X
Myrtaceae	*Xanthostemon sebertii* Guillaumin	Critically Endangered D ver 2.3	N	-	-	X
Orchidaceae	*Eulophia moratii* N. Hallé	Extinct ver 2.3	S	X	-	-
Orchidaceae	*Gunnarella aymardii* (N. Hallé) Senghas		N; S	X	-	-
Orchidaceae	*Zeuxine francii* Schltr.		N	X	X	-
Pandanaceae	*Pandanus verecundus* B. C. Stone		S	X	-	-
Paracryphiaceae	*Quintinia parviflora* (Schltr.) Schltr.	Critically Endangered D ver 2.3	S	-	X	-
Phellinaceae	*Phelline billardierei* Pancher ex Loes.		S	-	-	-
**Phellinaceae**	***Phelline microcarpa* Baill.**		N	-	-	X
Phyllanthaceae	*Phyllanthus balansaeanus* Guillaumin		S	-	-	-
Phyllanthaceae	*Phyllanthus baraouaensis* M. Schmid		N; S	-	-	X
Phyllanthaceae	*Phyllanthus calcicola* M. Schmid		N	-	-	-
Phyllanthaceae	*Phyllanthus conjugatus* M. Schmid	EN B2ab(iii)	S	X	-	X
Phyllanthaceae	*Phyllanthus dzumacensis* M. Schmid *		S	-	-	X
Phyllanthaceae	*Phyllanthus helenae* M. Schmid		N	-	-	-
Phyllanthaceae	*Phyllanthus jaubertii* Vieillard ex Guillaumin		N	-	-	-
Phyllanthaceae	*Phyllanthus koniamboensis* M. Schmid *		N	-	-	X
Phyllanthaceae	*Phyllanthus kouaouaensis* M. Schmid *		N	-	-	X
Phyllanthaceae	*Phyllanthus longeramosus* Guillaumin ex M. Schmid		S	-	-	-
Phyllanthaceae	*Phyllanthus luciliae* M. Schmid		N	-	-	-
Phyllanthaceae	*Phyllanthus memaoyaensis* M. Schmid		N	-	-	-
**Phyllanthaceae**	***Phyllanthus ningaensis* M. Schmid**		S	-	X	X
Phyllanthaceae	*Phyllanthus pilifer* M. Schmid		N	X	-	-
Phyllanthaceae	*Phyllanthus poueboensis* M. Schmid		N	-	-	-
Phyllanthaceae	*Phyllanthus pterocladus* S. Moore		N	-	-	X

Phyllanthaceae	*Phyllanthus tireliae* M. Schmid		N	X	-	X
Picrodendraceae	*Austrobuxus clusiaceus* (Baill.) Airy Shaw		S	-	-	X
Picrodendraceae	*Austrobuxus ovalis* Airy Shaw		N	-	X	-
Picrodendraceae	*Scagea oligostemon* (Guillaumin) Mc Pherson		S	-	X	X
Pittosporaceae	*Pittosporum aliferum* Tirel & Veillon	Endangered B1+2c ver 2.3	N	X	-	X
Pittosporaceae	*Pittosporum bouletii* Veillon & Tirel		N	-	-	-
Pittosporaceae	*Pittosporum brevispinum* Veillon & Tirel	EN A3ce	N	X	-	-
Pittosporaceae	*Pittosporum muricatum* Tirel & Veillon	Endangered B1+2c ver 2.3	S	X	-	X
Primulaceae	*Maesa jaffrei* M.Schmid	Critically Endangered B2ab(iii,v) ver 3.1	N	X	-	X
Primulaceae	*Rapanea belepensis* M.Schmid		N	-	-	-
Primulaceae	*Rapanea humboldtensis* M. Schmid		S	-	X	-
Primulaceae	*Rapanea ouameniensis* M. Schmid		S	-	-	-
Primulaceae	*Tapeinosperma baladense* Mez		N	-	-	-
Primulaceae	*Tapeinosperma boulindaense* M.Schmid, ined.		N	X	-	X
Primulaceae	*Tapeinosperma brevipedicellatum* M.Schmid, ined.		N	-	X	-
Proteaceae	*Beauprea congesta* Virot	Endangered D ver 2.3	S	-	X	-
Proteaceae	*Beauprea crassifolia* Virot	Vulnerable D2 ver 2.3	N	-	-	-
Proteaceae	*Stenocarpus heterophyllus* Brongn. & Gris	Endangered B1+2c ver 2.3	S	X	-	X
Proteaceae	*Stenocarpus villosus* Brongn. & Gris	Critically Endangered D ver 2.3	N	X	-	-
Proteaceae	*Virotia vieillardii* (Brongn. & Gris) P. H. Weston & A. R. Mast		N	X	-	-
Rubiaceae	*Atractocarpus aragoensis* Guillaumin		S	-	X	-
Rubiaceae	*Atractocarpus longestipitatus* Baill. ex Guillaumin		S	-	X	-
Rubiaceae	*Gea boulindaensis* Achille, ined. *		N; S	-	X	X
Rubiaceae	*Ixora aoupinieensis* Hoang & Mouly		N	X	X	-
Rubiaceae	*Ixora clarae* Mouly & Pisivin		S	-	X	-
Rubiaceae	*Psychotria bourailensis* Guillaumin		S	-	-	-
**Rubiaceae**	***Psychotria ferdinandi-muelleri* Guillaumin**		S	-	X	X
Rubiaceae	*Psychotria floribunda* (Montrouz.) Guillaumin		N	-	-	-
Rubiaceae	*Psychotria nekouana* (Baill.) Guillaumin		S	-	-	-

Rubiaceae	*Psychotria ouatilouensis* Guillaumin		S	-	-	-
Rubiaceae	*Psychotria stenophylla* Guillaumin *		S	-	-	X
Rubiaceae	*Thiollierea pachyphylla* (Guillaumin) Barrabé & Mouly, comb. ined.		S	-	X	-
Rutaceae	*Comptonella fruticosa* T.G.Hartley		N	X	-	X
Rutaceae	*Dutailliopsis gordonii* T.G Hartley		S	X	X	X
Rutaceae	*Dutaillyea amosensis* (Guillaumin) T.G.Hartley	Vulnerable D1 ver 2.3	N; S	X	-	-
Rutaceae	*Medicosma gracilis* T.G.Hartley *		N	-	-	X
Rutaceae	*Melicope leptococca* Baill. Ex Guillaumin		N	-	-	-
Rutaceae	*Melicope pedicellata* Guillamin		N; S	-	X	-
Rutaceae	*Neoschmidia calycina* T.G. Hartley		N	X	-	X
Rutaceae	*Sarcomelicope dognyensis* T.G.Hartley		N; S	-	-	-
Rutaceae	*Sarcomelicope pembaiensis* T.G.Hartley		N; S	-	-	-
Rutaceae	*Sarcomelicope sarcococca* (Baill.) Engl.		S	X	-	-
Salicaceae	*Casearia lifuana* Däniker		L	-	-	-
Salicaceae	*Homalium juxtapositum* Sleumer	Endangered B1+2c ver 2.3	N	X	-	X
Salicaceae	*Homalium polystachyum* (Vieill.) Briq.	Endangered B1+2c ver 2.3	N	X	-	X
Salicaceae	*Lasiochlamys cordifolia* Sleumer		N	-	X	-
Salicaceae	*Lasiochlamys manjeliana* Sleumer	Vulnerable D2 ver 2.3	N	-	-	-
Salicaceae	*Lasiochlamys rivularis* Sleumer		N; S	-	X	-
Salicaceae	*Lasiochlamys trichostemona* (Guillaumin) Sleumer	Lower Risk/conservation dependent ver 2.3	S	-	X	-
Salicaceae	*Xylosma boulindae* Sleumer	Vulnerable D1 ver 2.3	N; S	X	-	X
Salicaceae	*Xylosma capillipes* Guillaumin	Critically Endangered B1+2c ver 2.3	S	X	X	-
Salicaceae	*Xylosma grossecrenatum* (Sleumer) Lescot	EN B1ab(iii)+2ab(iii)	N	X	-	-
Salicaceae	*Xylosma kaalense* Sleumer	Vulnerable B1+2c ver 2.3	N	X	-	X
Salicaceae	*Xylosma lancifolium* Sleumer		N	-	-	-
Salicaceae	*Xylosma pininsulare* Guillaumin	Critically Endangered B1+2c ver 2.3	N; S	X	-	-
Santalaceae	*Amphorogyne staufferi* Markgr.		S	-	X	-
Santalaceae	*Exocarpos spathulatus* Schltr. & Pilg.		S	-	X	-
Sapindaceae	*Arytera nekorensis* H.Turner	Vulnerable B1+2c ver 2.3	N; S	X	-	-
Sapindaceae	*Cupaniopsis glabra* Adema	Endangered B1+2c ver 2.3	S	X	-	X

Sapotaceae	*Beccariella vieillardii* (Baill.) Swenson, Bartish & Munzinger		N; S	X	-	X
Sapotaceae	*Planchonella cauliflora* Munzinger & Swenson		N; S	-	-	-
Sapotaceae	*Planchonella koumaciensis* Aubrév. *		N	-	-	X
Sapotaceae	*Planchonella mandjeliana* Munzinger & Swenson		N	-	X	-
Sapotaceae	*Planchonella minutiflora* Munzinger & Swenson		N	X	-	-
Sapotaceae	*Planchonella roseoloba* Munzinger & Swenson		N	-	X	-
Sapotaceae	*Pycnandra bracteolata* Swenson & Munzinger		N	-	X	-
Sapotaceae	*Pycnandra caeruleilatex* Swenson & Munzinger ined.		S	-	-	-
Sapotaceae	*Pycnandra elliptica* Swenson & Munzinger ined.		S	-	-	-
Sapotaceae	*Pycnandra micrantha* (Beauvis.) Munzinger & Swenson, comb. ined.		N	-	-	-
Sapotaceae	*Pycnandra paucinervia* Swenson & Munzinger		N	-	X	-
Sapotaceae	*Pycnandra viridiflora* Swenson & Munzinger		N; S	-	-	-
Sapotaceae	*Pycnandra wagapensis* (Guillaumin) Swenson & Munzinger, comb. ined.		N; S	-	-	-
Simaroubaceae	*Soulamea cardioptera* Baill.		S	X	-	X
Solanaceae	*Solanum hugonis* Heine	EN B1ab(iii)+2ab(iii)	N	-	-	-
Stemonuraceae	*Gastrolepis alticola* Munzinger, McPherson, Lowry		S	-	X	-
Symplocaceae	*Symplocos gracilis* Brongn. & Gris		N	-	-	-
Violaceae	*Agatea veillonii* Munzinger		N	-	-	-
Winteraceae	*Zygogynum fraterculum* Vink		N	-	-	-

Narrow endemic species restricted to 3 locations

Species name in bold indicates that 100% of the populations are impacted by mines. Asterisks after the species name indicates that at least 50% of records are impact by mines. N: North province; S: South province. An « X » in the different columns indicates that species are protected by local legislation/located in a protected area/impacted by mining activities.

Family	Species	IUCN status	Province occurrence	Protected by local legislation	Populations located in a protected area	Populations impacted by mining activities
Acanthaceae	*Graptophyllum balansae* Heine		S	-	-	-
Alseuosmiaceae	*Platyspermation crassifolium* Guillaumin	Lower Risk/conservation dependent ver 2.3	S	-	X	-
Anacardiaceae	*Euroschinus aoupiniensis* M.Hoff	Vulnerable B1+2c ver 2.3	N	X	X	-
Apocynaceae	*Alstonia boulindaensis* Boiteau		N	X	-	-
Apocynaceae	*Alyxia poyaensis* (Boiteau) D.J.Middleton		N	X	-	X
Apocynaceae	*Alyxia torqueata* (Baill.) Guillaumin		N; S	X	-	X
Apocynaceae	*Cerberiopsis neriifolia* (S. Moore) Boiteau	Endangered B1+2c ver 2.3	S	X	X	-
Apocynaceae	*Marsdenia koniamboensis* Guillaumin *		N	-	-	X
Apocynaceae	*Marsdenia pseudoparsonsia* Guillaumin		N	-	-	X
Apocynaceae	*Melodinus reticulatus* Boiteau		N; S	X	-	X
Apocynaceae	*Parsonsia macrophylla* Pichon ex Guillaumin		N; S	-	X	X
Araliaceae	*Meryta lecardii* (R. Vig.) Lowry & F. Tronchet, ined.		N; S	-	X	X
Araliaceae	*Plerandra emiliana* (Baill.) Lowry, Plunkett & Frodin		N; S	-	X	X
Araliaceae	*Plerandra pachyphylla* (Harms) Lowry, Plunkett & Frodin		S	-	-	-
Araliaceae	*Polyscias dzumacensis* Lowry & Plunkett, ined *		S	-	X	X
Araliaceae	*Polyscias jaffrei* Lowry & Plunkett, ined. *		N	-	-	X
**Araliaceae**	***Polyscias scopoliae* (Baill.) Lowry**	Vulnerable B1+2c ver 2.3	N; S	-	-	X
Arecaceae	*Actinokentia huerlimannii* H. E. Moore	Lower Risk/conservation dependent ver 2.3	S	X	-	-
Arecaceae	*Chambeyronia lepidota* H.E. Moore		N	X	X	-
Arecaceae	*Clinosperma lanuginosa* (M.Schmid ex H.E.Moore) Pintaud & W.J.Baker		N	-	X	-
Arecaceae	*Kentiopsis oliviformis* (Brongn. & Gris) Brongn.	Endangered B1+2c ver 2.3	N	X	-	-
Arecaceae	*Kentiopsis pyriformis* Pintaud & Hodel		S	X	X	X
Argophyllaceae	*Argophyllum acinetochromum* Guillaumin		N	-	-	X
**Argophyllaceae**	***Argophyllum brevipetalum* Guillaumin**		S	-	-	X
Argophyllaceae	*Argophyllum grunovii* Zahlbr. *		S	-	X	X
Celastraceae	*Elaeodendron bupleuroides* (Guillaumin) R. H. Archer		S	-	X	-

Celastraceae	*Salaciopsis tiwakae* I. H. Müller		N	-	-	-
Chrysobalanaceae	*Hunga guillauminii* Prance	Vulnerable B1+2c ver 2.3	N	X	-	X
Chrysobalanaceae	*Hunga lifouana* (Däniker) Prance		L; N	-	-	-
Cunoniaceae	*Codia belepensis* H. C. Hopkin		N	X	-	-
Cunoniaceae	*Cunonia aoupiniensis* Hoogland	Vulnerable D2 ver 2.3	N	-	X	-
Cunoniaceae	*Pancheria ajiearoana* H. C. Hopkins, Pillon & J.Bradford *		N	-	-	X
Cunoniaceae	*Pancheria dognyensis* H. C. Hopkins, Pillon & J. Bradford		N; S	-	-	-
Cunoniaceae	*Pancheria mcphersonii* H.C.Hopkins, Pillon & J.Bradford		N	-	X	-
Cunoniaceae	*Pancheria multijuga* Guillaumin ex H.C.Hopkins & J.Bradford	Lower Risk/conservation dependent ver 2.3	S	-	X	-
Cunoniaceae	*Pancheria xaragurensis* H.C. Hopkins & Pillon, ined.		S	-	X	X
Cyperaceae	*Costularia neocaledonica* Rendle		S	-	X	-
Ebenaceae	*Diospyros margaretae* F. White	Vulnerable D1 ver 2.3	N; S	-	-	X
Elaeocarpaceae	*Elaeocarpus moratii* Tirel	Vulnerable D1 ver 2.3	N	-	X	-
Elaeocarpaceae	*Sloanea koghiensis* Tirel		S	-	X	-
Ericaceae	*Cyathopsis violaceo-spicata* (Guillaumin) Quinn		N	X	-	X
Euphorbiaceae	*Baloghia brongniartii* (Baill.) Pax *		N; S	-	X	X
Euphorbiaceae	*Baloghia neocaledonica* (S. Moore) Mc Pherson		S	-	-	X
**Euphorbiaceae**	***Bocquillonia goniorrhachis* Airy Shaw**		N	-	-	X
Euphorbiaceae	*Bocquillonia longipes* Mc Pherson	Endangered B1+2c ver 2.3	N	X	-	X
Euphorbiaceae	*Cleidion lochmios* McPherson	Vulnerable B1+2c ver 2.3	N	X	-	-
Euphorbiaceae	*Cleidion marginatum* McPherson	Vulnerable B1+2c ver 2.3	S	-	-	-
Euphorbiaceae	*Cleidion velutinum* McPherson		N	X	-	X
Fabaceae-Caesalpinioideae	*Storckiella neocaledonica* I.C. Nielsen, Labat & Munzinger		N; S	X	-	X
Lamiaceae	*Oxera crassifolia* Virot	Lower Risk/conservation dependent ver 2.3	S	-	X	-
Lauraceae	*Beilschmiedia neocaledonica* Kosterm. *		N; S	-	-	X
Lauraceae	*Endiandra poueboensis* Guillaumin		N	-	-	-
Malpighiaceae	*Stigmaphyllon mackeeanum* C.E. Anderson		N	-	-	X
Malvaceae	*Acropogon domatifer* Morat	Vulnerable D2 ver 2.3	N	-	X	-
Malvaceae	*Acropogon grandiflorus* Morat & Chalopin		N	-	X	-

Malvaceae	*Acropogon pilosus* Morat & Chalopin		N	-	-	-
Malvaceae	*Acropogon schistophilus* Morat & Chalopin		N	-	X	-
Malvaceae	*Corchorus neocaledonicus* Schlechter		N	-	-	X
Meliaceae	*Dysoxylum pachypodum* (Baill.) C. DC.	Critically Endangered D ver 2.3	N; S	X	-	-
Myrtaceae	*Eugenia balansae* Guillaumin		S	-	X	-
Myrtaceae	*Eugenia dagostinii* J.W.Dawson, ined.	EN A2ce	N; S	-	-	-
Myrtaceae	*Eugenia virotii* Guillaumin	Vulnerable B1+2c ver 2.3	N	X	-	X
Myrtaceae	*Kanakomyrtus myrtopsidoides* Guillaumin ex N.Snow		S	-	X	-
Myrtaceae	*Metrosideros paniensis* J.W.Dawson		N	-	X	-
Myrtaceae	*Syzygium boulindaense* J. W. Dawson		N	-	-	-
Myrtaceae	*Syzygium jaffrei* J. W. Dawson		N; S	-	X	X
Myrtaceae	*Syzygium koniamboense* J. W. Dawson *		N	-	-	X
Myrtaceae	*Syzygium kuebiniense* J. W. Dawson		S	-	-	-
Myrtaceae	*Syzygium meorianum* J. W. Dawson		N	-	-	-
Myrtaceae	*Syzygium pennelii* (Guillaumin) J. W. Dawson		N; S	-	-	-
Myrtaceae	*Syzygium poyanum* J. W. Dawson	VU B1ab(iii)+2ab(iii)	N; S	-	-	X
Myrtaceae	*Tristaniopsis vieillardii* Brongn. & Gris	Vulnerable B1+2c ver 2.3	S	-	-	X
Myrtaceae	*Xanthostemon ferrugineus* J. W. Dawson *		N	-	-	X
Myrtaceae	*Xanthostemon grisei* Guillaumin		N	-	-	-
Myrtaceae	*Xanthostemon gugerlii* Merr.		N	-	-	X
Nothofagaceae	*Nothofagus baumanniae* (Baum.-Bod.) Steenis	Lower Risk/conservation dependent ver 2.3	S	-	X	-
Orchidaceae	*Acianthus aegeridantennatus* N. Hallé		N; S	X	-	-
Orchidaceae	*Acianthus tenellus* Schltr.		N	X	-	X
Orchidaceae	*Bulbophyllum atrorubens* Schltr.		N	X	X	-
Orchidaceae	*Bulbophyllum keekee* N. Hallé		N; S	X	X	X
Orchidaceae	*Dendrobium cleistogamum* Schltr.		S	X	X	-
Orchidaceae	*Dendrobium munificum* (Finet) N. Hallé		S	X	-	-
Orchidaceae	*Liparis zosterops* N. Hallé		N	X	-	X
Orchidaceae	*Sarcochilus koghiensis* Schltr.		N; S	X	-	-
Pandanaceae	*Pandanus clandestinus* Stone	Lower Risk/conservation dependent ver 2.3	N	-	X	-

Paracryphiaceae	*Quintinia oreophila* (Schltr.) Schltr.		S	-	X	X
Paracryphiaceae	*Sphenostemon comptonii* Baker f.		N	-	X	-
Phyllanthaceae	*Phyllanthus boguenensis* M. Schmid		S	-	X	-
Phyllanthaceae	*Phyllanthus favieri* M. Schmid *		N	-	-	X
Phyllanthaceae	*Phyllanthus jaffrei* M. Schmid		N	X	-	X
Phyllanthaceae	*Phyllanthus mouensis* M. Schmid		S	-	X	X
Phyllanthaceae	*Phyllanthus polygynus* M. Schmid		N	X	-	X
Phyllanthaceae	*Phyllanthus vespertilio* Baill.		N	-	-	-
Picrodendraceae	*Austrobuxus alticola* McPherson		N	-	X	-
Poaceae	*Greslania montana* Balansa		S	-	X	-
Poaceae	*Oryza neocaledonica* Morat	En B1ab(iii)+2ab(iii)	N	X	-	-
Podocarpaceae	*Podocarpus decumbens* N.E.Gray		S	-	X	-
Primulaceae	*Rapanea katrikouensis* M. Schmid		N; S	-	-	-
Primulaceae	*Rapanea kuebiniensis* M. Schmid		S	-	-	X
Primulaceae	*Rapanea mcphersonii* M. Schmid		S	-	X	-
Primulaceae	*Rapanea nigricans* M. Schmid		N; S	-	-	X
Primulaceae	*Rapanea obovalifolia* M. Schmid		N	-	X	-
Primulaceae	*Tapeinosperma aragoense* Guillaumin		N	-	-	-
Primulaceae	*Tapeinosperma ateouense* M.Schmid, ined.		N	-	X	-
Primulaceae	*Tapeinosperma ellipticum* Mez		N	-	X	-
Primulaceae	*Tapeinosperma rubriscapum* Guillaumin		N; S	-	-	-
Primulaceae	*Tapeinosperma tchingouense* M.Schmid, ined. *		N	-	-	X
Proteaceae	*Stenocarpus dumbeensis* Guillaumin	Extinct ver 2.3	S	-	-	-
Rubiaceae	*Gea humboldtensis* (Guillaumin) Achille, ined.		S	-	X	X
Rubiaceae	*Ixora elisae* Mouly & Pisivin		L	-	-	-
Rubiaceae	*Ixora longiloba* Guillaumin		N; S	-	X	X
Rubiaceae	*Psychotria brachylaena* (Baill.) Guillaumin		N	-	-	X
Rubiaceae	*Psychotria ianthina* Guillaumin *		N	-	-	X
Rubiaceae	*Psychotria pulchrebracteata* Guillaumin		N	-	X	-
Rubiaceae	*Tarenna lifouana* (Däniker) Jérémie		L	-	-	-

Rutaceae	*Medicosma exigua* T.G.Hartley		N	X	-	X
Rutaceae	*Oxanthera brevipes* Stone	Vulnerable B1+2c ver 2.3	N	X	-	X
Rutaceae	*Oxanthera neocaledonica* (Guillaumin) Tanaka	Endangered B1+2c ver 2.3	N	X	-	X
Salicaceae	*Casearia coriifolia* Lescot & Sleumer	Lower Risk/conservation dependent ver 2.3	N; S	X	X	X
Salicaceae	*Casearia kaalaensis* Lescot & Sleumer	Endangered B1+2c ver 2.3	N	X	-	-
Salicaceae	*Homalium mathieuanum* (Vieill.) Briq.	Endangered B1+2c ver 2.3	N	X	-	-
Salicaceae	*Homalium rubiginosum* (Vieill.) Warb. *	Vulnerable B1+2c ver 2.3	N	-	-	X
Salicaceae	*Xylosma tuberculatum* Sleumer *	Vulnerable D2 ver 2.3	N	-	-	X
Santalaceae	*Exocarpos clavatus* Stauffer		S	-	X	X
Sapindaceae	*Cupaniopsis chytradenia* Radlk.		N	-	-	-
**Sapindaceae**	***Cupaniopsis megalocarpa* Adema**		S	-	-	X
Sapindaceae	*Elattostachys incisa* Radlk.		N; S	-	-	-
Sapotaceae	*Polanchonella saligna* S. Moore		N	-	-	-
Sapotaceae	*Pycnandra multipetala* (Vink) Swenson & Munzinger, comb. ined.		S	-	X	-
Sapotaceae	*Pycnandra neocaledonica* (S.Moore) Vink		N	-	-	-
Simaroubaceae	*Soulamea pelletieri* Jaffré & Fambart *		S	-	-	X
Winteraceae	*Zygogynum acsmithii* Vink		S	-	-	-
Winteraceae	*Zygogynum cristatum* Vink *	Vulnerable B1+2c ver 2.3	N; S	-	-	X
