# Supplementary material for: Conservation Priorities in a Biodiversity Hotspot: Analysis of Narrow Endemic Plant Species in New Caledonia
Source: PLoS One. 2013 Sep 18;8(9):e73371. doi: 10.1371/journal.pone.0073371 (PMC3776834; doi:10.1371/journal.pone.0073371)
Supplement: Appendix S3 — Sites with a high likelihood of containing NES that were not identified as HPNE from the distributional records alone. (PDF) [file pone.0073371.s003.pdf]

Appendix 3: Sites with a high likelihood of containing NES that were not identified as HPNE from the distributional records alone. Potential reasons for the difference between the distributional data and the model output are outlined below.

Group 1

SITE	REASONS FOR MISMATCH
1 : Low altitude and dry areas of the West Coast	Anthropic degradation.
2 : Houailou river	Anthropic degradation.
3 : Kouaoua	Anthropic degradation.
4 : Canala	Anthropic degradation.
5 : St Vincent Bay surroundings	Anthropic degradation.
6: Between Port Bouquet and Ouinée	Under sampled, mine and fire degradations. Sampling needed at low and medium altitudes in the Neuménie, To N'Deu, Nimbo, Comboui and N'Goye valleys.
7 : Rivière Bleue	7 NES recorded (just under the threshold of 8 NES for a HPNE).
8 : Cap Pouareti	Anthropic degradation.

Group 2

10 : Tnèda, Ga Wenem, Gan Tuétai	Under sampled in the high valleys, especially on volcano-sedimentary substrates above 600 m and on ultramafic soil above 900 m.
11: Gaitada, Pwailatimbé, Ticahouiane, Pomongoa, Wöwélang Bwali	Under sampled (some old samples). Fires degradations on the east and north east slopes. The summit and the west and south slopes (starting at 600 m) should be investigated.
12 : Goro Atuwaa, Taji	Under sampled. Sampling is needed in contact areas between volcano-sedimentary and ultramafic substrates (between 400 and 800 m and at high elevation above 1000 m). Access is difficult.
13 : Goro Até, Grota	Under sampled. Sampling is needed in contact areas between volcano-sedimentary and ultramafic substrates.
15 : Mont Arago, Le Sphinx	Under sampled. Contact areas of ultramafic and volcano-sedimentary substrates especially on the summit of "Le Sphinx" should be looked at. Slopes all around shelter dense vegetation between 700 and 1000 m that need to be investigated.
16 : Karavéyaa, Mé Ixaburu, Karacu	Under sampled, especially around the Méré river.
17 : Mont Canala, Mont Nakada, Xûkinea	Under sampled (some old samples). Sampling is needed in the forest areas on the east and south east slopes between 700 and 1100 m. The other areas are impacted by fires (summit and north slopes).

Group 3

23 : Mé Maoya, Keiyouma	Under sampled. North and north east slopes are degraded. All the others areas between 700 and 1500 m need to be sampled. Access is difficulte. **This area should be considered as a priority.**
24 : Mé Adéo	Under sampled. Sampling is needed in contact areas between volcano-sedimentary and ultramafic substrats between 600 and 1000 m.
26 : Koungouhaou Nord and South	Under sampled and degraded by fires up to the summit.
Dent de St Vincent	Under sampled (some old samples). The highest areas, between 900 and 1400 m are interesting and not well known.
Bwa Bwi	Under sampled. Dense forests between 700 and 1200 m totally un-sampled. **This area should be considered as a priority.**
Mont Ningua	Under sampled (sampling occurred during this study).
Mont Cidoa	Under sampled.
Mont Ouin	Under sampled, especially between 800 and 1300 m.
Mont Tonta	Under sampled, especially relics of forests between 800 and 1000 m. Degradation by fires.
